# Body Size Reductions in Nonmammalian Eutheriodont Therapsids (Synapsida) during the End-Permian Mass Extinction

**DOI:** 10.1371/journal.pone.0087553

**Published:** 2014-02-03

**Authors:** Adam K. Huttenlocker

**Affiliations:** Department of Biology and Burke Museum of Natural History and Culture, University of Washington, Seattle, Washington, United States of America; University of Birmingham, United Kingdom

## Abstract

The extent to which mass extinctions influence body size evolution in major tetrapod clades is inadequately understood. For example, the ‘Lilliput effect,’ a common feature of mass extinctions, describes a temporary decrease in body sizes of survivor taxa in post-extinction faunas. However, its signature on existing patterns of body size evolution in tetrapods and the persistence of its impacts during post-extinction recoveries are virtually unknown, and rarely compared in both geologic and phylogenetic contexts. Here, I evaluate temporal and phylogenetic distributions of body size in Permo-Triassic therocephalian and cynodont therapsids (eutheriodonts) using a museum collections-based approach and time series model fitting on a regional stratigraphic sequence from the Karoo Basin, South Africa. I further employed rank order correlation tests on global age and clade rank data from an expanded phylogenetic dataset, and performed evolutionary model testing using Brownian (passive diffusion) models. Results support significant size reductions in the immediate aftermath of the end-Permian mass extinction (ca. 252.3 Ma) consistent with some definitions of Lilliput effects. However, this temporal succession reflects a pattern that was underscored largely by Brownian processes and constructive selectivity. Results also support two recent contentions about body size evolution and mass extinctions: 1) active, directional evolution in size traits is rare over macroevolutionary time scales and 2) geologically brief size reductions may be accomplished by the ecological removal of large-bodied species without rapid originations of new small-bodied clades or shifts from long-term evolutionary patterns.

## Introduction

Body size is an important biological trait that affects organismal fitness and imparts a strong influence on ecological, physiological, and life history attributes of species [1]–[4]. As such, size and size-related traits have received considerable attention from paleobiologists interested in characterizing the tempo and mode of morphological evolution in extinct groups. Famous examples include ‘Cope's rule,’ an apparent tendency for animal lineages to achieve larger sizes over time [5]–[7], and more recently the ‘Lilliput effect,’ short-term size reductions in animal taxa following mass extinction events [8]–[10]. In the case of Cope's rule, workers historically attributed patterns of body size increase to non-random selection within lineages over geologic time, an intuitive conclusion when surveying some of the best examples of macroevolutionary transitions in the fossil record [11]–[14]. However, several studies suggest that many of these important paleontological transitions (e.g., expansions in dinosaur body sizes) were in fact influenced by Brownian processes of trait evolution, random tendencies for lineages to diffuse through size space with increasing disparity [7], [15]–[18]. Considerably less attention has been paid to the underlying influences of Lilliput-type patterns and mass extinctions. Background patterns of origination/extinction during the Neogene indicate lower turnover rates (i.e., long species durations) in smaller-bodied mammals, possibly due to their higher reproductive rates, large population sizes, and frequent sleep-or-hide burrowing behavior compared to large mammals [19], [20]. Nevertheless, size-related extinction risk remains controversial [21]–[23], and the supposed chaotic and indiscriminate nature of mass extinctions, as in the end-Cretaceous and end-Permian mass extinctions, calls into question whether these dynamics are applicable to rapid, global-scale catastrophes [24].

Though originally reserved for within-lineage size decreases in survivor taxa [8], the term ‘Lilliput’ has been repurposed as a pattern that may encompass a number of other underlying processes, such as differential extinction of large-bodied taxa and rapid diversifications of new small-bodied clades following mass extinctions [9], [10], or a combination of these [25]. These patterns have been particularly well documented in Triassic marine invertebrate communities following the end-Permian mass extinction [9], [25]–[28] and anecdotally in terrestrial vertebrates of the earliest Triassic *Lystrosaurus* Assemblage Zone, Karoo Basin of South Africa [29]–[31]. However, the extent to which the end-Permian mass extinction influenced body size reductions and possible departures from background patterns of body size evolution are imprecisely known. This is in part because the temporal and phylogenetic components of body size evolution before and after the extinction have never been compared. Here, I use time series analysis and a reference phylogeny of Permian through Triassic eutheriodont therapsids (therocephalians and cynodonts) to better understand the tempo and mode of body size evolution in the therapsid forerunners of mammals during a mass extinction interval. Notably, therocephalian eutheriodonts originated at relatively large sizes during the Middle Permian (estimated maximum skull length, ∼40 centimeters), while many of their Triassic relatives, including some cynodonts and baurioid therocephalians, achieved comparatively diminutive sizes (estimated skull length ∼2.5–3.0 centimeters in brasilodontids and early Mammaliaformes). Therocephalians and cynodonts provided a rare opportunity to study post-extinction body size shifts in terrestrial vertebrates due to their range of sizes, relative generic richness in earliest Triassic faunas, and differential survivorship of the end-Permian extinction [32], [33]. I identify short-term reductions in maximum and mean body sizes during the earliest Triassic, and suggest that geologic patterns of size reductions in lowermost Triassic rocks do not reflect rapid diversifications of new small-bodied clades or changes in evolutionary patterns following the extinction. Rather, observed short-term body size reductions arose from interplay between a backdrop of increasing size disparity and a temporary culling of large-bodied therapsid predators during the end-Permian extinction. Institutional abbreviations are listed in Appendix S1.

## Approach

### Stratigraphic (Non-phylogenetic) Patterns

Non-random extinction patterns may be discernible with regard to some organismal traits, especially when studied in the context of existing patterns of trait evolution during background intervals. Non-phylogenetic investigations of selectivity in Cretaceous-Paleogene marine invertebrates have suggested that mass extinction may bring about changes in selectivity patterns that reset the stage for biotic recoveries. This departure from normal (background) selectivity patterns has been termed “non-constructive selectivity” [24]. Patterns of ecomorphological change have been well studied in stratigraphic series, particularly in Permo-Triassic and Cretaceous-Paleogene marine faunas [24], [34], [35], but similar approaches have only been recently applied to body size in terrestrial vertebrates. For example, Wilson [36] identified significant post-extinction body size reductions in a succession of Cretaceous-Paleogene mammals from northeastern Montana, and suggested constructive selectivity against large, dietary specialists possibly due to depressed primary productivity during that time. Similar patterns have been suggested in earliest Triassic terrestrial disaster faunas, including selection for small-bodied generalists with drought-tolerant food sources and fossorial or semi-aquatic habits [29], [30], [32], [37]. I propose that investigations of selectivity during the end-Permian mass extinction should address firstly whether non-random patterns are discernible and, if so, whether they deviate from pre-existing patterns of trait evolution. First, I test whether maximum and mean body sizes decreased significantly in the earliest Triassic *Lystrosaurus* Assemblage Zone (hereafter ‘AZ’) using non-phylogenetic time series and statistical comparisons of size distributions between temporally successive eutheriodont assemblages. Second, I chose to implement time series modeling and rank correlation tests to evaluate whether reductions, if present, signified transient shifts or longer-term directional size shifts both across the extinction and during pre-extinction intervals. A transient shift following mass extinction (an expectation of Lilliput-type patterns) would be indicated by short-term shifts in stratigraphic series without a significant mean step change across the entire sampled interval (or during pre-extinction intervals).

#### Geologic controls and sampling

Sampling of non-isotaphonomic intervals and unevenness of sampling may bias the quality and size controls of the present sample. These can include but are not restricted to available outcrop area, collection intensity, and sediment coarseness and sorting, which should be considered given the comparatively small sizes observed in the Triassic *Lystrosaurus* AZ. Recent assessments of geologic controls on paleodiversities have found potential associations between outcrop area and taxonomic richness of pre-extinction assemblages, but the outlying *Lystrosaurus* AZ exhibits unexpectedly modest richness that is unexplainable by outcrop area alone [38]–[41], in spite of intense collecting effort and large numbers of specimens from this interval. A comparison of the diversities of therocephalians and cynodonts from these assemblages yields comparable results (Appendix S2). Also, differences in grain sizes may produce differential size-sorting in fossil assemblages, where coarse sediments deposited in high-energy environments may preferentially preserve larger, more robust skeletal remains. However, the fine-grained floodplain deposits and paleosols of the Permian Balfour Formation give way to rhythmically-bedded sheet sandstone bodies and thick channel sands and conglomerates of the Triassic Katberg Formation, producing a general coarsening upward in the Permo-Triassic boundary sequence of the Karoo. It is therefore unlikely that this coarsening upward has produced a bias in small-bodied eutheriodonts in the *Lystrosaurus* AZ. While taphonomic and collecting biases are important considerations, I suggest that they would have had limited impacts on diversity and body size shifts observed in the *Lystrosaurus* AZ.

### Phylogenetic Patterns

Size shifts in post-extinction faunas, if present, may have been underpinned to some extent by stochastic processes of trait evolution. Long-term negative shifts observed throughout the sampled interval may indicate background patterns of trait evolution (e.g., general tendencies toward body size reductions). Size shifts may alternatively represent rapid diversifications of new clades whose success was associated with the trait in question (in this case, small body size). Rapid diversification of small-bodied taxa following extinction is one explanation for Lilliput-type patterns, although few examples have been demonstrated [25], [42]. Nevertheless, an inadequate understanding of phenotypic evolution during background intervals hinders comparisons of post-extinction dynamics. It is therefore informative to compare stratigraphic patterns of trait evolution to those patterns inferred by cladogenetic branching events. This approach allows investigators to identify directionality, characterize the timing and location of important cladogenetic events, and distinguish those features from more general (long-term) features of phenotypic evolution during background intervals. I chose to implement ancestor-descendant tests in order to (1) evaluate the relative frequency at which smaller descendants evolved from larger ancestors in a reconstructed lineage of ancestor-descendant pairs, and to (2) identify the branches (internodes) in which those negative shifts occurred. Diversification rate shifts were also assessed to determine whether post-extinction radiations of small, Triassic taxa during biotic recovery could have contributed to observed shifts in size distributions of eutheriodont faunas. Finally, I evaluated the fit of phylogenetic models to determine whether trait evolution was best approximated by directional, ‘stabilized,’ or initially rapid but decelerating (‘early burst’) models, or a Brownian motion model. These approaches establish a baseline of pre-extinction evolutionary dynamics, and help to determine whether selectivity during the extinction was constructive (i.e., whether it mirrored background patterns) or non-constructive in nature. I argue here that extinctions were largely size selective, but were constructive, dependent upon existing patterns of trait evolution, and did not result in shifts from long-term phylogenetic patterns in the study group.

## Methods 1: Stratigraphic Patterns

### Database Assembly

#### Specimen sampling

I assembled a database of specimen measurements and stratigraphic occurrences for 72 species of Permian and Triassic therocephalians and cynodonts. The majority of measurements were taken directly from museum specimens, but data for some non-African taxa that were inaccessible during the study period were taken from the literature. Permission to access collections was obtained from all visited institutions prior to the study period. By incorporating data from museum specimens, I was able to assess variation within species, including maximum sizes, rather than representative samples from the literature. A rigorous, collections-based survey therefore allows previous literature-based assessments of body size evolution in Permo-Triassic therapsids to be improved upon [17], [18]. Taxa were assigned to one or more stratigraphic bins, which mainly follow the biozonation of the Karoo Basin, the best-sampled and most complete terrestrial Permo-Triassic boundary succession in the world 43,44. The Karoo biozonation includes distinct assemblage zones identified by their fossil tetrapod associations: *Eodicynodon*, *Tapinocephalus*, *Pristerognathus*, *Tropidostoma*, *Cistecephalus*, *Dicynodon*, *Lystrosaurus*, and *Cynognathus* AZs. Additional global data, collected to distinguish non-regional geologic and phylogenetic trends in all sampled Permian and Triassic taxa, covered a wider stratigraphic interval so that late Middle Triassic (Ladinian) and Late Triassic (Carnian, Norian, Rhaetian) global standard stages were also included as distinct bins. The sample was limited to Permian and Triassic genera only, and to specimens having well-preserved skulls for complete skull length measurements. Several Early Jurassic nonmammaliaform cynodonts from the Karoo (e.g., *Diarthrognathus*, *Pachygenelus*, *Tritylodon*) and those lacking complete skulls were therefore excluded from the dataset. The resulting database included 347 complete cranial specimens and 72 species of Permian through Triassic therocephalians and cynodonts from the Karoo Basin and contemporaneous basins. Sources for occurrence data, including voucher specimens and absolute dates, are provided in Appendix S3. Stratigraphic nomenclature and dates for Middle-to-Late Triassic basins follow Abdala et al. [45], Abdala and Ribeiro [46], Irmis et al. [47] and references cited therein.

#### Measurements

From the complete database, 57 measurements were retained for time series analysis of the Karoo assemblages, and 72 measurements were retained for rank order analyses of the global dataset representing the maximum basal skull lengths of the 72 sampled taxa from the stratigraphic interval of their first appearance (therocephalians: N = 37; cynodonts: N = 35). Basal skull length (BSL), measured from the tip of the snout to the occiput in millimeters (mm), was chosen as a proxy for body size for several reasons. Firstly, many of the fossil taxa examined here are best represented by cranial material, which is more accessible than postcranial elements and is relatively diagnostic for nonmammalian therapsids (contributing to the accuracy of specimen identifications). Moreover, investigation of BSL provides direct comparisons to other published studies using similar proxies. Skull length also correlates well with other linear skeletal measurements in therapsids such as femur length [17], [18]. Measurements were recorded to the nearest mm using digital Mitutoyo calipers. For each taxon, the ‘max lnBSL’ represents the natural log of BSL for the largest measurable individual observed within a given interval. By assessing only the maximum lnBSL of each taxon (rather than taxon averages), these measures permitted both a conservative estimate of size reductions from one interval to the next while also reducing the effects of differences in relative abundance.

### Evaluation of Time Series Models

Time series data represent a sequence of observations repeated over elapsed time, and can be conceptualized in a paleontological context as ancestor-descendant relationships (in as much as they assume that traits in daughter populations are a product of evolutionary changes from preceding populations, lineages, or clades) [48],[49]. I performed time series analysis to evaluate regional patterns of body size evolution in a stratigraphic succession of therocephalians and cynodonts from the Permian-Triassic Beaufort Group, Karoo Basin, South Africa. First, I evaluated the probability that the body size samples from the Late Permian (*Cistecephalus* and *Dicynodon* AZs) and earliest Triassic (*Lystrosaurus* AZ) were drawn from equivalent distributions using a Kolmogorov-Smirnov (K-S) test. Then, I evaluated the likelihood that observed negative shifts in size distributions (if present) represent a random evolutionary walk (rather than a long-term directional trend) by assessing the record of body size evolution over eight stratigraphic bins of the Karoo succession. Mean lnBSL was estimated for each of eight stratigraphic bins by taking the average of the maximum known lnBSL for each taxon within a given bin, therefore representing a per taxon average (rather than an average of all specimens, which could otherwise be skewed by taxa with unusually high abundances). In contrast to the rank order correlation tests, which examined only size at first appearance (discussed below), taxa whose ranges spanned multiple assemblage zones were included in each bin in which they occurred in order to capture more fully the variance within each bin. Mean sizes were estimated in each bin for all eutheriodonts, as well as separately for therocephalians and cynodonts in order to evaluate the pervasiveness of potential trends among the therapsid subclades.

Mean lnBSL and disparity (as measured by variance) were estimated from the database for each Karoo biozone (average bin duration ≈3 Myr) and imported into the ‘R’ version 2.15.1 statistical environment using the ‘paleoTS’ package [48], [49]. The ‘paleoTS’ package permited evaluation of temporal patterns of body size evolution across the Karoo succession, using several models that fall into two general categories: non-directional and directional. Non-directional models implemented by the ‘paleoTS’ package include unbiased random walk (or ‘URW’) and stasis. URW models evolution as a volatile Brownian process with observable step variance but no mean step change, whereas stasis models evolution with modest deviations that are uncorrelated and normally distributed around the trait mean [49]. Directional evolution is modeled as a generalized random walk (‘GRW’) having a positive or negative mean step change. The relative fit of non-directional and directional evolutionary models was evaluated using likelihood methods and goodness-of-fit tests. My expectation is that, if short-term (Lilliput-type) size reductions are a unique signature of the Triassic *Lystrosaurus* AZ, then longer-term size reductions should not be manifested in the Karoo time series, with non-directional models best fitting the data (i.e., URW and stasis). If, on the other hand, body size evolution was driven by longer-term trends beginning in the Permian, then a directional model with a negative mean step change should best fit the data, thereby refuting the rapid and short-term perturbation predicted by the Lilliput effect.

The relative fit of the three models (URW, stasis, and GRW) was assessed using Akaike's information criterion corrected for small sample sizes (AICc). AICc is an information loss metric that is used to compare models both through their goodness-of-fit and model complexity (i.e., the number of adaptable model parameters). AICc diminishes the problem of overfitting complex models, and is widely used for small paleontological and neontological datasets. Akaike weights (‘AW’) are estimated by converting the individual AICc scores for each candidate model into a proportional value representing the model's relative support (with the sum of the values adding to 1.0 across all models); the model with the highest weight represents that with the least information loss (and, thus, the most desirable model). A more complete description of AICc and its application to time series and phylogenetic model fitting can be found in Hunt and Carrano [49].

### Rank Order Correlations

Rank order statistics provide additional tests for identifying directional trends in ranked paleontological data (in this case age ranks, clade ranks and patristic distances) [16], [50]–[53]. ‘Age rank’ represents an ordered numerical representation of the stratigraphic bin in which a taxon first appeared, particularly useful where estimated durations of bins and positions of taxa therein are only imprecisely known. Rank order correlations were performed here to assess the relationship between size and age rank, as well as the relationships between size, clade rank and patristic distance (Methods 2). Non-parametric assessments of binned age ranks permitted comparisons to results of the clade rank analyses, which used similar rank order tests. Stratigraphic bins were divided into 13 ranks (average duration ≈5 Ma), increased from eight in the Karoo-only time series to accommodate late-Middle and Late Triassic taxa. Each taxon in the global dataset was assigned an age rank, according to the ranked stratigraphic bin in which it first appeared, and an associated maximum lnBSL within that bin. Correlations between size and age rank were assessed using Spearmans' rho (*ρ*) and Kendall's tau (*τ*) rank order tests. These tests allowed potentially positive or negative trends in the global dataset to be evaluated as a monotonic function (the Spearman's rank correlation describing the strength of a monotonic association, and Kendall's tau assessing the probability of observing concordant and discordant pairs among ranked variables). Importantly, unlike their parametric equivalent, Pearson's product moment correlation, the tests are robust even when the association is not linear. Three separate data subsets were analyzed to evaluate directional size trends in eutheriodonts (*N* = 72) and therocephalian (*N* = 37) and cynodont (*N* = 35) subclades from the Middle Permian through Late Triassic. For Eutheriodontia and its subclades, I evaluated *ρ* and *τ* coefficients for negative trends in two ways, assessing 1) data subsets that included taxa only having first appearances in the Permian and 2) the total Permian-Triassic dataset. By examining Permian-only and excluding Triassic originations, I was able to assess the extent to which general trends in the total Permian-Triassic dataset were influenced by new size distributions of post-extinction taxa (versus pre-existing or ‘background’ patterns of trait evolution). Trends were considered only weakly significant if both coefficients were negative but non-significant for one of the tests.

## Results 1: Stratigraphic Patterns

### Time Series Model Fitting

**Figure 1:**
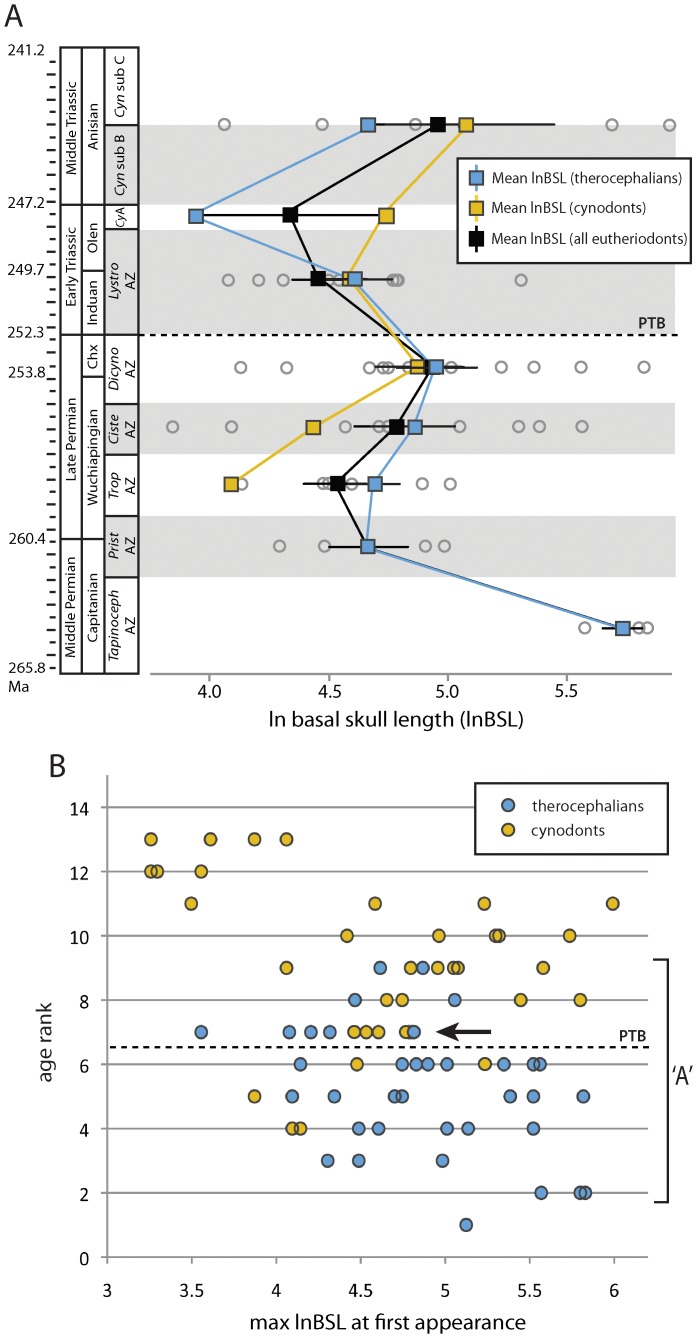
Karoo time series and age rank results for observed geologic distributions of maximum basal skull length (lnBSL). A , Regional patterns of trait evolution in Karoo eutheriodonts (black) (therocephalian subclade = blue; cynodont subclade = gold) fit a non-directional model throughout the Karoo succession (see Table 1). Squares represent clade averages (with 95% confidence intervals), gray circles represent maximum known sizes for each species recorded in a given stratigraphic interval (Ma, millions of years ago). **B**, Spearman's rank correlation tests on global age rank data (used for the phylogenetic sampling) reveal a weak negative trend in species maximum sizes (*ρ* = −0.261; *p* = 0.025) with a short-term bottleneck in size maxima in the Early Triassic *Lystrosaurus* AZ (arrow). Permian-Triassic boundary (PTB) represented by dashed line.

My evaluation of stratigraphic (i.e., non-phylogenetic) patterns failed to identify long-term negative trends in eutheriodont body size evolution, instead showing a short-term reduction in mean sizes in the Triassic *Lystrosaurus* AZ. Visual inspection of the Karoo time series data suggests a transient decrease in mean size from the Late Permian *Dicynodon* AZ to the *Lystrosaurus* AZ, which rebounded subsequently in the *Cynognathus* AZ (Fig. 1). Kolmogorov-Smirnov tests on size distributions from the *Lystrosaurus* AZ suggest significant decreases when compared against the Late Permian *Cistecephalus* and *Dicynodon* AZs (therocephalians: *D* = 0.61, *p*<0.001; cynodonts: *D* = 0.38, *p* = 0.021). A binomial test on the Karoo dataset using the *binom.test* function of paleoTS [49] found no systematic tendency for decreases in mean size of eutheriodonts over the Karoo succession, suggesting that this geologically abrupt shift was not part of an observable long-term trend. This may, however, reflect the poor ability of the statistical test to reject the null hypothesis of a random walk, given the small number of stratigraphic bins available. I therefore used the small sample size version of Akaike's Information Criterion (AICc) to evaluate the relative fit of directional (generalized random walk or ‘GRW’) and non-directional (unbiased random walk or ‘URW’ and evolutionary stasis) candidate models, but non-directional models found the best support as size differences accrued with noticeable trait variance but with a step mean of zero (Table 1). Observed size decreases in the *Lystrosaurus* AZ therefore cannot be attributed to a long-term directional trend when evaluated as a time series.

**Table 1:** Time series model fitting of body size evolution in Karoo therocephalians and cynodonts.

	GRW			URW			Stasis		
	AICc	AW	*μ, σ^2^*	AICc	AW	*σ^2^*	AICc	AW	*θ, ω*
**Eutheriodontia_Karoo_**	19.848	0.025	−0.029, 0.042	*14.601*	*0.349*	*0.045*	**13.435**	**0.625**	**4.794, 0.077**
**Therocephalia_Karoo_**	*16.669*	*0.159*	*−0.038, 0.000*	**14.540**	**0.462**	**0.031**	*14.942*	*0.378*	*4.818, 0.079*
**Cynodontia_Karoo_**	16.937	0.033	0.052, 0.000	**11.495**	**0.503**	**0.008**	*11.658*	*0.464*	*4.702, 0.023*

GRW, generalized random walk (directional); URW, unbiased random walk;

AICc, Akaike's Information Criterion corrected for small sample size; AW, Akaike weight;

*μ*, step mean; *σ^2^*, step variance; *θ*, trait mean; *ω*, trait variance;

bold = best model; italics = non-negligible model (AW ≥1/8 best model).

### Age Ranks and Rank Order Correlations

Rank order correlations between age rank and size at first appearance, on the other hand, may indicate a weak negative trend for some groups such as therocephalians, which originated at their largest sizes in the Permian (Fig. 1B, Table 2). This correlation, however, is likely influenced both by a lack of originations of new large Triassic therocephalians (pulling higher age ranks into the lower end of size space) and the ranking and evaluation of data as a monotonic function. When Triassic taxa are removed from the analysis, correlation coefficients are only weakly negative and non-significant, indicating an inability of the tests to identify long-term negative trends from Permian size distributions. This result may be owed to the somewhat smaller sample size of the Permian-only data set (27 versus 37 therocephalians), but most Permian bins reflect the persistence of large-bodied forms and high disparity (variance) up until the Permo-Triassic boundary. Permian cynodonts, however, were difficult to evaluate due to their low taxonomic richness during the Permian and, consequently, small sample sizes. In summary, Early Triassic eutheriodont faunas were on average smaller-bodied than in preceding Permian assemblages. However, Permian stratigraphic trends alone provide little evidence of long-term declines in mean body sizes.

**Table 2:** Results of rank correlations, Spearman's rho (*ρ*) and Kendall's tau (*τ*), for global age ranks.

Age rank *v.* lnBSL		*N* taxa	ρ	*p*(ρ)	τ	*p*(τ)
**Eutheriodontia**	(Permian taxa only)	32	−0.089	0.621	−0.054	0.693
**Therocephalia**	(Permian taxa only)	27	−0.153	0.443	−0.089	0.559
**Cynodontia**	(Permian taxa only)	5	–	–	–	–
**Eutheriodontia**	(Permian + Triassic)	72	**−0.261**	**0.025**	**−0.173**	**0.037**
**Therocephalia**	(Permian + Triassic)	37	**−0.370**	**0.023**	**−0.254**	**0.035**
**Cynodontia**	(Permian + Triassic)	35	−0.265	0.115	−0.113	0.362

## Methods 2: Phylogenetic Patterns

### Reference Cladogram, Clade Ranks and Patristic Distances

**Figure 2:**
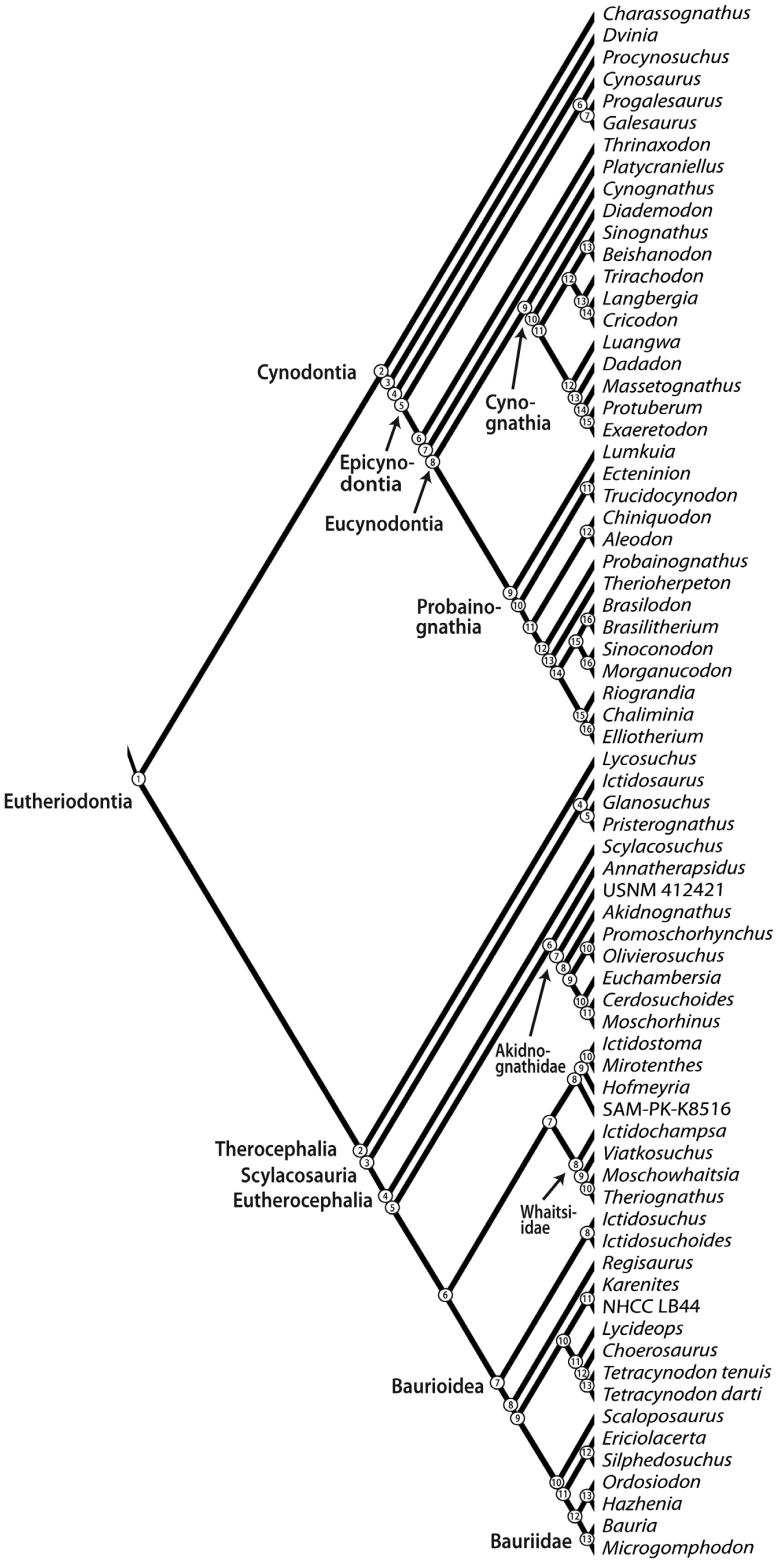
Complete reference cladogram with nodes numbered for estimation of patristic distances. Sources include: therocephalians, Huttenlocker [54], Huttenlocker et al. [33], Sigurdsen et al. [55]; cynodonts, Hopson & Kitching [56], Abdala et al. [45], Sidor & Hancox [57], Ranivoharimanana et al. [60], Oliveira et al. [58], Liu & Olsen [59].

**Figure 3:**
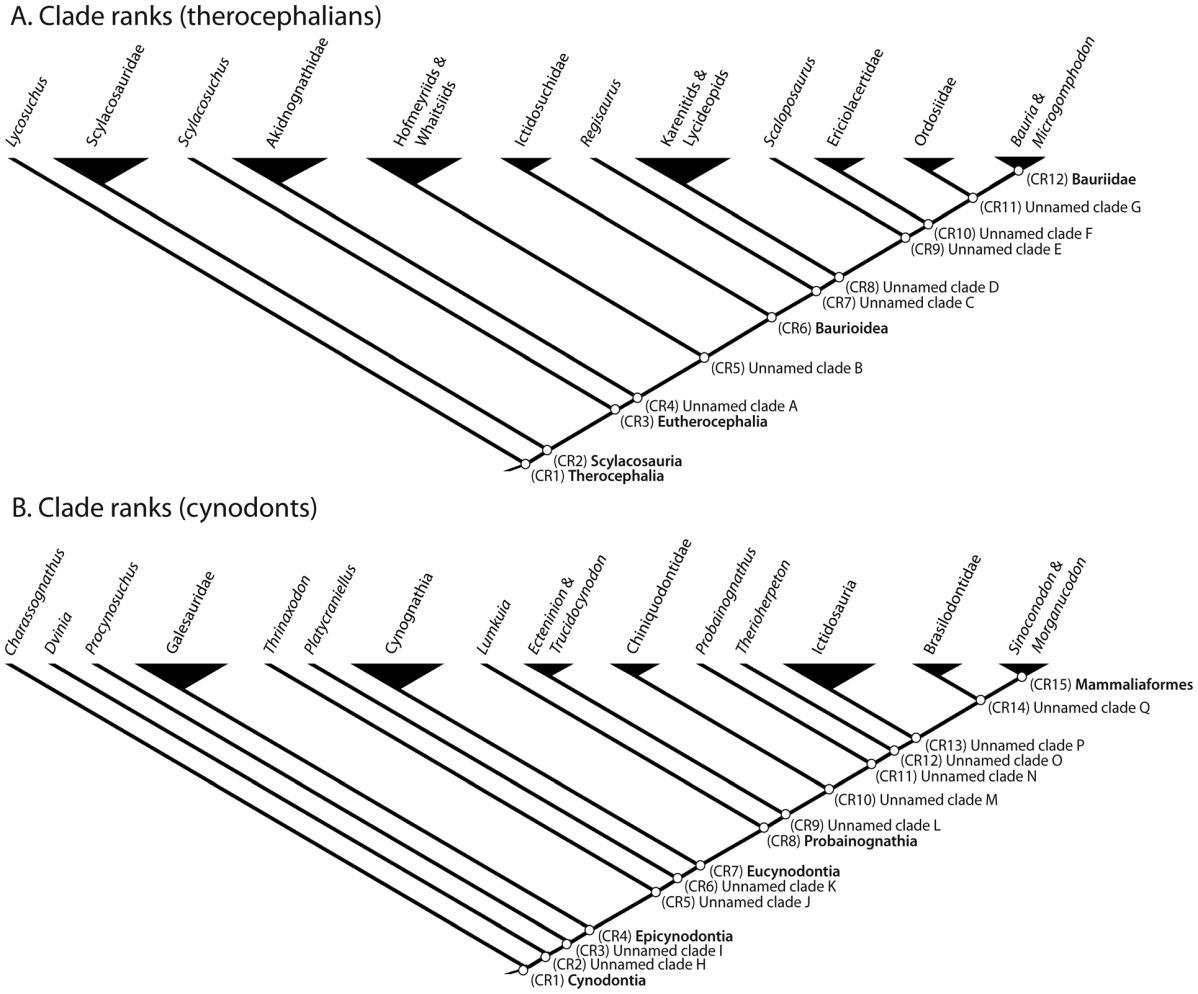
Ranking of side-branches within the two major subgroups: Therocephalia (A) and Cynodontia (B) (simplified from reference cladogram in **Fig. 2**).

Cladistic data were also ranked to permit the evaluation of directional trends in size data with respect to relative branching events in a reference cladogram (Fig. 2). I constructed an informal supertree primarily from the literature, although some new taxa and specimens are included here in an expanded analysis of therocephalian therapsid relationships updated from [33], [54], [55]. Inclusion of these additional taxa facilitated the most comprehensive phylogenetic sample of therocephalian therapsids to date, updated from Sigurdsen et al. [55] (see Dataset S1). I follow several sources for cynodont phylogeny, which has received greater attention in the literature [43], [56]–[60]. ‘Clade ranks’ (CR) were assigned based on the number of nodes passed along the backbone of the tree (e.g., Cynodontia to Mammaliaformes) without incorporating branching events of clades off the primary axis. This metric has provided a useful tool for assessing long-term evolutionary patterns along the primary axis of the tree with respect to early mammaliaforms [50], [61]. ‘Patristic distances’ (PD) represented the number of nodes passed through from the hypothetical ancestor at the base of the tree to each of the tips [52]. Therocephalians and cynodonts were analyzed separately for correlations with CR because these two main subclades replicate CR values (Fig. 3). PD values bear no such constraint and were estimated directly from the complete reference phylogeny in Figure 2. Rank order correlation coefficients were estimated from Permian-only and combined Permian-Triassic datasets as in the aforementioned age rank analysis. Statistical results are presented in Table 3.

**Table 3:** Results of rank correlations, Spearman's rho (*ρ*) and Kendall's tau (*τ*), for clade ranks and patristic distances.

Clade rank *v.* lnBSL		*N* taxa	ρ	*p*(ρ)	τ	*p*(τ)
**Therocephalia**	(Permian taxa only)	27	**−0.608**	**0.001**	**−0.488**	**<0.001**
**Cynodontia**	(Permian taxa only)	4	–	–	–	–
**Therocephalia**	(Permian + Triassic)	37	**−0.622**	**<0.001**	**−0.484**	**<0.001**
**Cynodontia**	(Permian + Triassic)	34	**−0.371**	**0.032**	−0.234	0.064

Patristic distance *v.* lnBSL		*N* taxa	ρ	*p*(ρ)	τ	*p*(τ)
**Eutheriodontia**	(Permian taxa only)	31	−0.135	0.467	−0.136	0.306
**Therocephalia**	(Permian taxa only)	27	**−0.508**	**0.007**	**−0.407**	**0.005**
**Cynodontia**	(Permian taxa only)	4	–	–	–	–
**Eutheriodontia**	(Permian + Triassic)	71	**−0.281**	**0.019**	**−0.189**	**0.022**
**Therocephalia**	(Permian + Triassic)	37	**−0.546**	**<0.001**	**−0.417**	**<0.001**
**Cynodontia**	(Permian + Triassic)	34	−0.192	0.279	−0.093	0.439

### Ancestor-Descendant Tests

Ancestor-descendant tests were performed as an extension of the CR analyses in order to assess tendencies toward negative versus positive size changes in the phylogenetic tree between reconstructed ancestor and descendant nodes [15], [16]. Whereas CR analyses allowed interpretations only from observed tip data, this analysis relies on squared-change parsimony to reconstruct the hypothetical traits of ancestors (which cannot be observed, only estimated). Ancestral states were estimated using squared-change parsimony in Mesquite version 2.0 [62]. For each nested clade, I then manually estimated the mean step change, the number of positive and negative changes between internodes (internodes having zero change were not counted), and the skewness, providing measures of tendency that could contribute to directional evolution in the tree. Results are presented in Table 4.

**Table 4:** Summary of ancestor-descendant step changes in the therocephalian and cynodont trees (ancestral states reconstructed using squared-change parsimony).

Eutheriodontia	BSL_anc_	lnBSL_anc_	Mean Δ	+	–	Skewness
**Therocephalia**	170	5.135	−0.006	9	25	1.213
Scylacosauria	170	5.135	−0.006	9	25	1.199
Eutherocephalia	162	5.087	−0.023	8	23	1.314
Unnamed clade A	151	5.017	−0.022	8	22	1.282
Unnamed clade B	140	4.941	−0.032	6	16	1.273
Baurioidea	135	4.905	−0.081	3	11	1.431
Unnamed clade C	128	4.852	−0.095	2	10	1.493
Unnamed clade D	120	4.787	−0.098	2	9	1.474
Unnamed clade E	56	4.025	0.077	2	3	1.379
Unnamed clade F	53	3.970	0.110	2	2	1.180
Unnamed clade G	153	5.030	−0.270	1	1	–
**Cynodontia**	61	4.110	0.016	15	15	0.173
Unnamed clade H	61	4.110	0.016	15	15	0.165
Unnamed clade I	60	4.094	0.018	15	14	0.153
Epicynodontia	48	3.871	0.026	15	13	0.093
Unnamed clade J	92	4.521	−0.026	13	13	−0.124
Unnamed clade K	88	4.477	−0.025	13	12	−0.129
Eucynodontia	219	5.389	−0.064	12	12	−1.370
Probainognathia	112	4.718	−0.045	7	5	−1.531
Unnamed clade L	123	4.812	−0.058	6	5	−1.402
Unnamed clade M	134	4.897	−0.096	4	5	−1.091
Unnamed clade N	82	4.406	−0.065	3	4	−1.464
Unnamed clade O	33	3.496	0.075	3	3	1.609
Unnamed clade P	30	3.401	0.109	3	2	1.509
Unnamed clade Q (incl. Mammaliaformes)	28	3.332	0.032	1	1	–

Ancestral basal skull length estimates (BSL_anc_) are in mm; mean change refers to log change between ancestor-descendant pairs;

+, number of internodes having a positive step change; –, number of internodes having a negative step change.

### Diversification Rate Shifts

To assess the hypothesis that shifts in mean body size represented post-extinction radiations of small-bodied eutheriodont clades, a phylogeny was constructed using all studied taxa in Figure 2 and tested for variations in diversification rates using the topology-based method of Chan and Moore [63]. The software SymmeTREE version 1.1 was used to evaluate the topological distribution of taxic diversity across branches in the reference phylogeny (i.e., its symmetry) and to identify regions of the tree that contribute to significant imbalance in diversity. This operation was performed by calculating the Δ_1_ and Δ_2_ statistics, functions of two likelihood ratios that represent the probability of observing a shift in diversification rate along the internal branch of a three-taxon tree (composed of a local outgroup and two basalmost subclades of the ingroup). For each node, the ratios compared the likelihood of yielding the observed distribution of diversity under an equal-rates Markov branching model to that under a two-rate model. Importantly, the inclusion of non-contemporaneous clades sampled from disjunct stratigraphic bins may violate an assumption of the equal-rates Markov model by heterogeneously sampling diversity through time [64], [65]. Therefore, five trees were subsampled from the reference phylogeny, representing a cross-section of diversity for each of the five best sampled assemblage zones spanning the Permian-Triassic transition: *Tropidostoma*, *Cistecephalus*, *Dicynodon*, *Lystrosaurus*, and *Cynognathus* AZs. Following Botha-Brink and Angielczyk [65], trees were pruned such that they only included taxa and ghost lineages present within the given stratigraphic bin. The trees were then evaluated in SymmeTREE to identify diversification rate shifts in post-extinction clades (e.g., bauriids; cynognathians or other eucynodonts) or in clades that diversified before the extinction (e.g., eutherocephalians; basal cynodonts).

### Evaluation of Phylogenetic Models

Finally, stochastic models of Brownian evolution were fitted to observed patterns of body size evolution in a comprehensive global phylogeny of eutheriodonts, permitting a marriage of temporal and topology-based methods. Phylogenetic model fitting was performed in R using ‘ape’ and ‘geiger’ packages [49]. The global reference phylogeny was exported into Newick notation and zero length branches that constrained ghost lineages of their sister clades were augmented using the smoothing distribution of the *date.phylo()* function (http://www.graemetlloyd.com/methdpf.html) [66]. Skull length data were imported and joined to individual taxon labels (corresponding to 71 tip taxa present on the tree), then assigned to the body size vector. Using ‘geiger,’ I evaluated the relative fit of four phylogenetic models in order to approximate the mode of body size evolution observed on the tree: Brownian motion (BM), Brownian motion with trend (BM_T_), Ornstein-Uhlenbeck (OU), and early burst Brownian motion (EB). BM models evolution as a process of passive diffusion (equivalent to an unbiased random walk); BM_T_, models evolution as a generalized random walk having a positive or negative mean step change (equivalent to a ‘directional trend’); OU models trait evolution as a constrained process approaching some optimal value (sometimes equated to ‘stabilizing selection’); and EB approximates a Brownian process in which the rate of trait evolution exponentially decreases (‘decays’) over time. Models were fitted to the data using ‘geiger,’ and likelihood ratio tests were performed to determine whether multiparameter models should be selected over the null BM model. Goodness-of-fit tests implementing AICc were also used to evaluate the candidate models as in the time series analysis.

## Results 2: Phylogenetic Patterns

### Cladogenetic Branching

**Figure 4:**
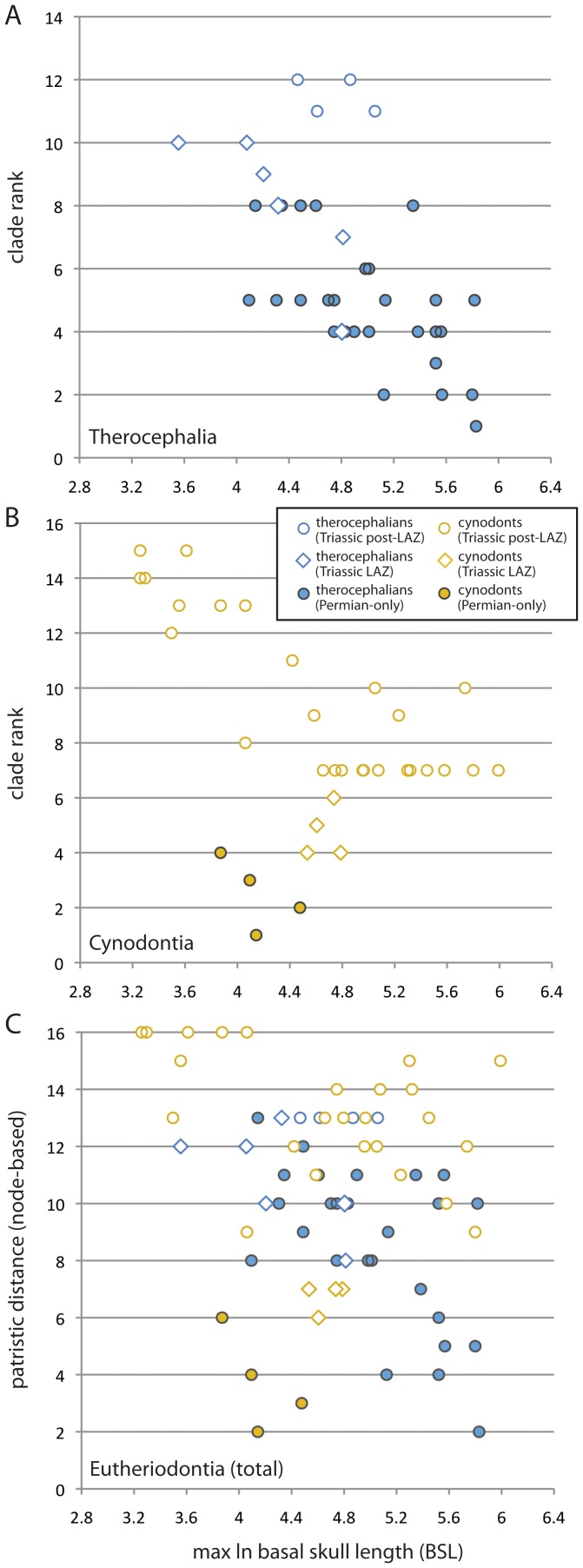
Spearman's rank correlation tests on clade rank (A, B) and patristic distance (C) versus maximum basal skull length for the global dataset of Middle Permian through Late Triassic eutheriodonts. Solid circles represent Permian taxa (therocephalian subclade = blue; cynodont subclade = gold), open diamonds represent Early Triassic *Lystrosaurus* Assemblage Zone (LAZ) taxa, and open circles represent Triassic post-LAZ taxa.

Cladistically-informed analyses were better able to detect long-term body size trends than stratigraphic data alone (Fig. 4; Table 3), with some groups expanding into smaller sizes relatively early in their phylogenetic histories and continuing in more deeply nested clades. Size exhibits a strong negative correlation with clade rank in therocephalians (*ρ* = −0.622, *p*<0.001; *τ* = −0.484, *p*<0.001) and a weakly negative correlation in cynodonts (*ρ* = −0.371, *p* = 0.032; *τ* = −0.234, *p* = 0.064). The same result is true for therocephalians whether analyzed with or without Triassic taxa, indicating that size reductions began in relatively shallowly-nested nodes in the tree (i.e., early-diverging groups which include Permian taxa) and continued into the Triassic. Correlations with patristic distance have lower statistical support, but are still significant for Permian-only and Permian-Triassic therocephalians indicating the influence of therocephalians on the overall pattern. I carried out ancestor-descendant tests following the method of Carrano [16] to supplement the rank correlation tests and to assess the tendency and relative frequency of negative size changes between reconstructed ancestor-descendant nodes. The results in Table 4 also indicate a strong tendency toward size reductions in therocephalians, with the number of ancestor-descendant decreases outnumbering increases by as much as three to five times. Cynodonts, which originated at relatively small sizes, showed no such tendency with the number of size decreases approximately equaling the number of increases.

### Diversification Rate Shifts

**Figure 5:**
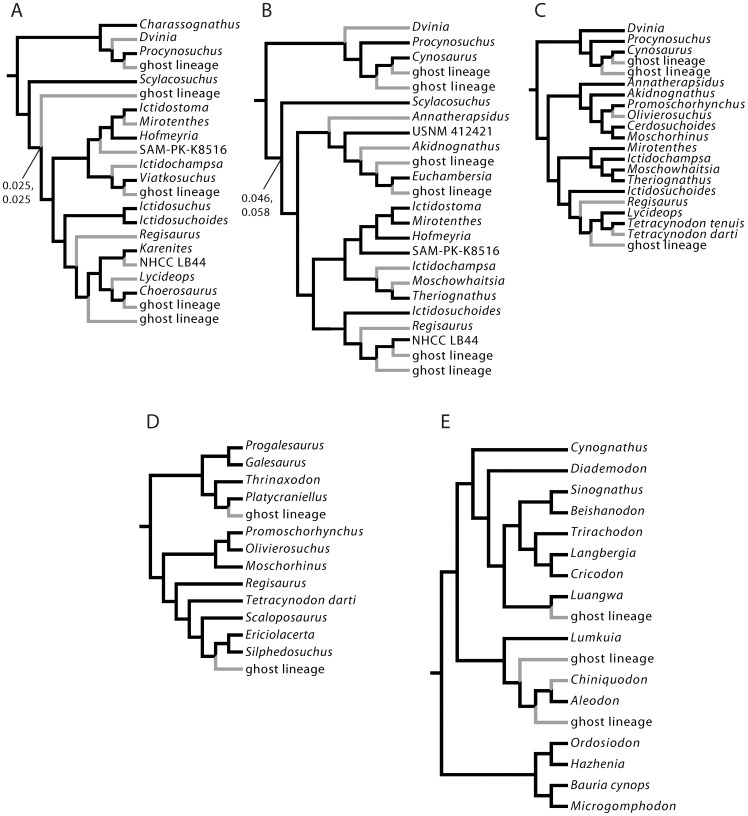
Cladograms subsampled from five stratigraphic bins for analysis of diversification rate shifts. A , *Tropidostoma* Assemblage Zone. **B**, *Cistecephalus* Assemblage Zone. **C**, *Dicynodon* Assemblage Zone. **D**, *Lystrosaurus* Assemblage Zone. **E**, *Cynognathus* Assemblage Zone. Gray branches represent ghost lineages for terminal taxa or clades that appear in a succeeding stratigraphic interval. Numbers at nodes represent *p*-values near or below 0.05 for the Δ_1_ (top) and Δ_2_ (bottom) statistics. Significant shifts are observed along the branch between the node of Eutherocephalia and non-*Scylacosuchus* eutherocephalians for the *Tropidostoma* AZ subsample (A), and a marginally significant shift is observed along the branch between Eutheriodontia and Eutherocephalia for the *Cistecephalus* AZ (B).

Results of rank order correlation and ancestor-descendant tests indicate that body sizes decreased on average in progressively deeper nested therocephalian subclades, supporting the prior expectation that body size shifts were not necessarily influenced by post-extinction diversity shifts (although they may have been associated with earlier diversifications). Accordingly, no significant rate shifts were detected in tree topologies constructed from Triassic *Lystrosaurus* or *Cynognathus* AZ diversity partitions, suggesting that either sampling is too poor to detect a shift or that the impact of the extinction on diversification rates in eutheriodonts was limited. The only significant diversification rate shift occurred at the early-Late Permian divergence of Eutherocephalia (Fig. 5A,B), a result that is consistent with the relatively high rates of per capita origination and extinction in Late Permian eutherocephalians [33], [67]. This early diversification rate shift also mirrors previous findings in stratigraphically contemporaneous bidentalian dicynodonts [65]. Alternatively, the high diversity of therapsids in the Late Permian *Tropidostoma* and *Cistecephalus* AZs could be a sampling artifact [39]. Nevertheless, no shift is detected in the well-sampled *Lystrosaurus* AZ following the extinction.

### Phylogenetic Model Fitting and Body Size Evolution

**Figure 6:**
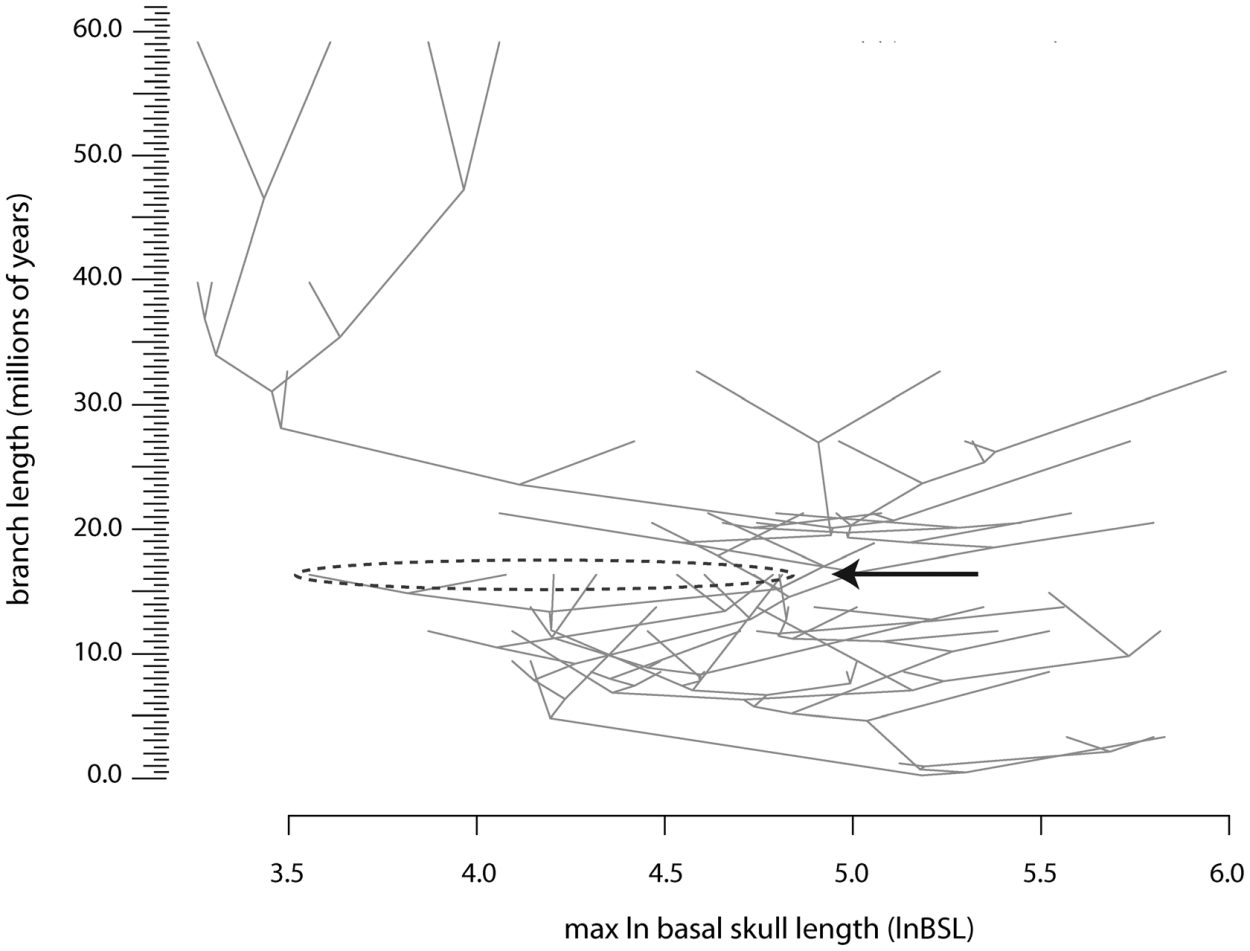
Traitgram of body size evolution in a global sample of Permian through Triassic eutheriodonts, approximating an ‘early burst’ Brownian process (EB) of trait evolution. Arrow denotes location of *Lystrosaurus* Assemblage Zone taxa along the time-axis. Graphic created using the *traitgram()* function in ‘picante.’

Goodness-of-fit tests found that active ‘directional’ (BM_T_) and ‘stabilizing’ (OU) models fit poorly when compared to other candidate models of passive, Brownian motion evolution (BM and EB). Likelihood ratio tests and comparisons of Akaike weights indicate that EB best approximates the mode of body size evolution across the global dataset of eutheriodonts (Figure 6; Table 5). Body size evolution was better approximated by the null BM model when therocephalians were analyzed separately, indicating that derived cynodont subclades strongly influenced the decay rate parameter for the EB model. In particular, late Middle and Late Triassic cynodont clades (e.g., stem-mammaliaforms and other probainognathians) that include members from outside of the Karoo succession became more constrained in size space with decreasing variance over time, but not other lineages that lived in the immediate aftermath of the end-Permian extinction in the Karoo. In short, reductions in sizes of *Lystrosaurus* AZ eutheriodonts were likely to have been underpinned by passive processes, rather than long-term directional trends or constraints on evolutionary modes during the end-Permian extinction.

**Table 5:** Phylogenetic model fitting results.

	BM			BM_T_			OU			EB		
	AICc	AW	*β*	AICc	AW	*β,μ,θ*	AICc	AW	*β,α*	AICc	AW	*β,r*
**Eutheriodontia_total_**	*113.72*	*0.276*	*0.05*	*115.61*	*0.107*	*0.05,0.01,5.15*	*113.41*	*0.270*	*0.06,0.02*	**112.79**	**0.346**	**0.09,−0.03**
**Therocephalia_total_**	**51.49**	**0.439**	**0.05**	*53.48*	*0.162*	*0.05,0.01,5.28*	*52.51*	*0.263*	*0.06,0.04*	*53.85*	*0.134*	*0.05,−0.006*
**Cynodontia_total_**	*58.32*	*0.118*	*0.05*	60.48	0.049	0.05,0.01,4.11	59.00	0.068	0.06,0.03	**54.99**	**0.764**	**0.17,−0.08**

Model selection based on comparison of corrected AICs and Akaike weights (AW) for Brownian motion (BM), Brownian motion with trend (BM_T_), Ornstein-Uhlenbeck (OU), and early burst (EB) models;

AICc, Akaike's Information Criterion corrected for small sample size; AW, Akaike weight;

*β*, BM rate parameter; *μ*, step mean; *θ*, trait mean; *α*, constraint parameter; *r*, decay rate;

bold = best model; italics = non-negligible model (AW ≥1/8 best model).

## Discussion

**Figure 7:**
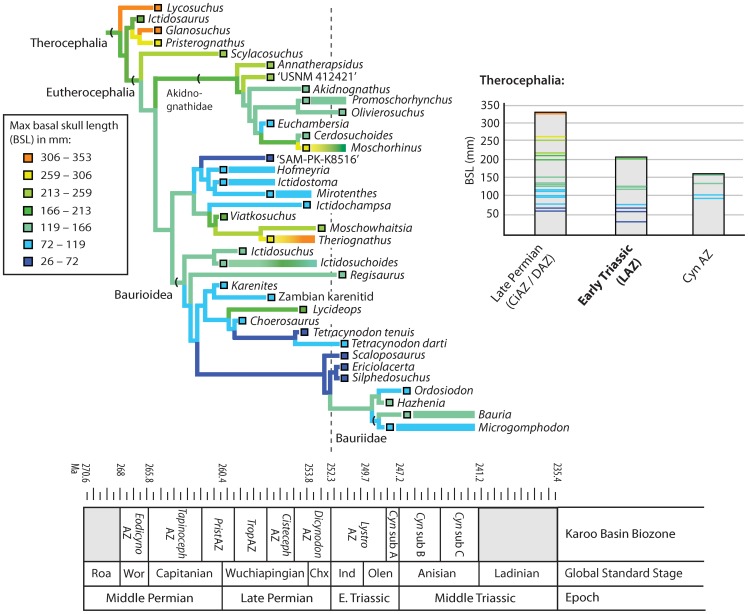
Ancestral state reconstructions for basal skull length in Permian and Triassic therocephalians. Colored horizontal bars indicate ranges of tip taxa spanning more than one stratigraphic bin. Note that significant decreases in mean sizes of major subgroups across the Permian-Triassic boundary (dashed line) were associated with the loss of large-bodied taxa and the survival of pre-existing small-bodied taxa or clades. Abbreviations: CiAZ, *Cistecephalus* Assemblage Zone; CynAZ, *Cynognathus* Assemblage Zone; DAZ, *Dicynodon* Assemblage Zone; LAZ, *Lystrosaurus* Assemblage Zone.

**Figure 8:**
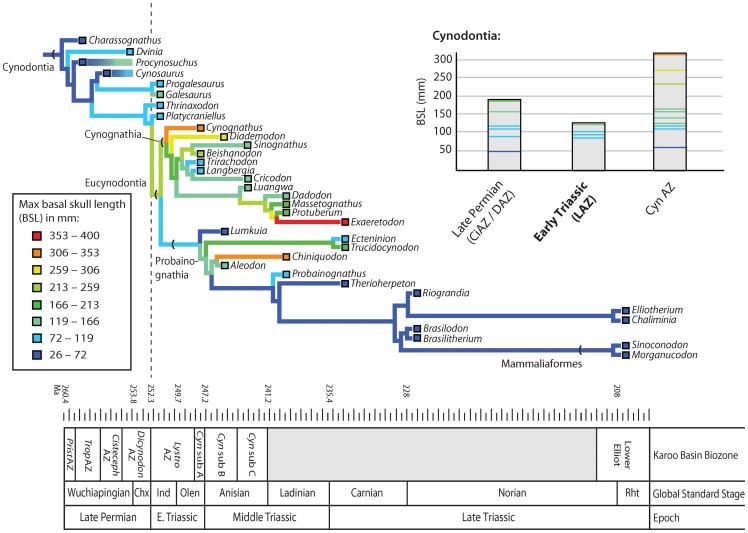
Ancestral state reconstructions for basal skull length in Permian and Triassic cynodonts. Colored horizontal bars indicate ranges of tip taxa spanning more than one stratigraphic bin (e.g., *Procynosuchus*, *Cynosaurus*). Permian-Triassic boundary indicated by dashed line. Abbreviations: CiAZ, *Cistecephalus* Assemblage Zone; CynAZ, *Cynognathus* Assemblage Zone; DAZ, *Dicynodon* Assemblage Zone; LAZ, *Lystrosaurus* Assemblage Zone.

Body size evolution before and immediately after the end-Permian extinction cannot demonstrably be classified as active or “driven” sensu McShea [15], because size increases were generally associated with as many or more decreases and with expanding size disparity. Moreover, short-term reductions in mean and maximum sizes in the earliest Triassic *Lystrosaurus* AZ were not associated with changes in these background patterns of Brownian evolution, but were instead a product of the temporary ecological removal of medium to large-bodied predators (in addition to anagenetic size reduction in at least one predator lineage, *Moschorhinus* [68]). In spite of its devastating ecological impacts, short-term shifts during the end-Permian extinction had negligible long-lasting effects on subsequent patterns of body size evolution in eutheriodont therapsids. Summary diagrams of eutheriodont body size evolution are provided in Figures 7 and 8, showing frequent size reductions beginning in the Permian with occasional increases in some lineages (contributing to increased disparity), and an overall drop in body size distributions in the Triassic *Lystrosaurus* AZ. Though in conflict with traditional views of the fossil record, this finding corroborates more recent suggestions that large-scale patterns of trait evolution rarely yield active, directional trends [17], [18], [48]. Negative shifts were likely passive and, in the case of therocephalians, may underscore a fundamental asymmetry in size evolution: that size decreases can often occur with higher frequency than increases due to physiological and/or environmental constraints on size [69]. The success of small-bodied lineages may therefore represent a more general feature of size evolution operating during background intervals, thereby representing a case of constructive selectivity as recently noted in Mesozoic mammals during the end-Cretaceous extinction [36], [70]. Early cynodonts apparently do not mirror the therocephalian pattern given the present sampling. However, it is noteworthy that they exhibited unusually small sizes relatively early in their evolutionary history, remained small for much of the Late Permian and earliest Triassic, and radiated into a wide range of sizes that included large-bodied forms only after the extinction of other large theriodont predators (e.g., *Theriognathus*, *Moschorhinus*, and gorgonopsians which were excluded in the present analysis). An unexpected result is the protracted and increasingly constrained evolutionary rates in late Middle to Late Triassic cynodonts. The factors influencing their constrained phenotypic evolution are beyond the scope of the present analysis, but may include intrinsic factors (developmental canalization) or extrinsic selective pressures (competition with larger-bodied archosaurs or other emerging clades) that merit future study.

The sudden and short-lived nature of Early Triassic size reductions is compatible with some ecological explanations for Lilliput effects. Suggested mechanisms driving Lilliput patterns have included 1) within-taxon size reductions in relict lineages, 2) extinction of large taxa, and/or 3) post-extinction radiations of small taxa [9], [10]. The phylogenetic models evaluated here do not support a post-extinction radiation of small taxa. In fact, several small-bodied *Lystrosaurus* AZ taxa and clades have Permian ghost lineages (Figs. 7, 8), implying preferential survivorship of these small-bodied lineages. Unfortunately, this latter scenario is difficult to assess because the small sample size of surviving tetrapod genera within the Karoo (only four observed records) is not amenable to the desired statistical tests (e.g., logistic regression of size-specific survivorship). At least one large-bodied eutheriodont, *Moschorhinus*, exhibited within-lineage size reductions across the extinction boundary, implying that the extinction also had the potential to act upon microevolutionary processes in a constructive manner [68]. Similar body size reduction characterizes the Permian and Triassic species of the dicynodont *Lystrosaurus* [65], [71].

This interplay of Lilliput mechanisms parallels that of some Permian-Triassic marine invertebrate clades in some respects, but differs in others. Global surveys of marine gastropods suggest strong within-lineage size decreases as well as size-selective extinction [26]. Earliest Triassic foraminifera from South China provide evidence of all three underlying processes [25], although within-lineage effects drive global size distributions of foraminifera most strongly [42]. Notably, within-lineage decreases in lingulid brachiopods underscore hitherto unexplored ecophysiological effects, as they were associated with more frequent interruptions to growth and slower overall growth rates (perhaps in response to ocean acidification and hypercapnia) [28]. Within-lineage size decreases are observed in some nonmarine tetrapods (e.g., *Moschorhinus* and *Lystrosaurus*), but more general shifts toward smaller terrestrial vertebrate taxa were likely accentuated by the ecological removal of large-bodied Permian species (although small size did not necessarily ensure survival). Recent surveys of bone histology in these Permian-Triassic survivor taxa provide little evidence that Triassic therapsids grew more slowly than their Permian predecessors, and in some cases they may have grown more quickly or over shorter durations [65], [68]. More data on the life histories of earliest Triassic terrestrial vertebrates will shed further light on the physiological and ecological underpinnings of these large-scale patterns and selectivity during the end-Permian biotic crisis.

## Supporting Information

Appendix S1 **List of institutional abbreviations.**
(PDF)

Appendix S2 **Outcrop area and sampled diversity of Karoo Basin therocephalians and cynodonts.**
(PDF)

Appendix S3 **Age and clade rank data for therocephalian and cynodont subclades. Basal skull length measurements (BSL) are in mm.**
(PDF)

Dataset S1 **Phylogenetic methods and supporting NEXUS file.**
(PDF)
