# Supplementary material for: Body Size Reductions in Nonmammalian Eutheriodont Therapsids (Synapsida) during the End-Permian Mass Extinction
Source: PLoS One. 2014 Feb 3;9(2):e87553. doi: 10.1371/journal.pone.0087553 (PMC3911975; doi:10.1371/journal.pone.0087553)
Supplement: Appendix S1 — List of institutional abbreviations. (PDF) [file pone.0087553.s001.pdf]

Appendix S1

Appendix S1. List of institutional abbreviations.

AMNH, American Museum of Natural History, New York; **BP**, Evolutionary Studies Institute (previously Bernard Price Institute for Palaeontological Research), University of Witwatersrand, Johannesburg; **CGS**, Council for Geoscience, Pretoria (former Geological Survey field numbers); **GSN**, Geological Survey of Namibia, Windhoek; **FMNH**, Field Museum of Natural History, Chicago; **IVPP**, Institute of Vertebrate Paleontology and Paleoanthropology, Chinese Academy of Sciences, Beijing; **MCN,** Museu de Ciências Naturais, Fundação Zoobotânica do Rio Grande do Sul, Porto Alegre; **MCZ**, Harvard University Museum of Comparative Zoology, Cambridge; **MGB**, Museo Guido Borgomanero, Mata; **MVP**, Museu Vicente Pallotti, Santa Maria; **NHCC**, National Heritage Conservation Commission, Lusaka; **NHMUK**, Natural History Museum, London; **NMQR**, National Museum, Bloemfontein; **PIN**, Paleontological Institute, Moscow; **PKUP**, Peking University Paleontology Collections, Beijing; **PULR**, Universidad Nacional de La Rioja; **PVL**, Fundación Miguel Lillo, Tucumán; **PVSJ**, Museo de Ciencias Naturales, Universidad Nacional de San Juan, San Juan; **RC**, Rubidge Collection, Wellwood, Graaff-Reinet; **SAM**, Iziko South African Museum, Cape Town; **TM**, Ditsong National Museum of Natural History (former Transvaal Museum), Pretoria; **UCMP**, University of California Museum of Paleontology, Berkeley; **UFRGS**, Universidade Federal do Rio Grande do Sul, Porto Alegre; **USNM**, National Museum of Natural History, Washington, D.C.
