# Supplementary material for: Body Size Reductions in Nonmammalian Eutheriodont Therapsids (Synapsida) during the End-Permian Mass Extinction
Source: PLoS One. 2014 Feb 3;9(2):e87553. doi: 10.1371/journal.pone.0087553 (PMC3911975; doi:10.1371/journal.pone.0087553)
Supplement: Appendix S2 — Outcrop area and sampled diversity of Karoo Basin therocephalians and cynodonts. (PDF) [file pone.0087553.s002.pdf]

Appendix S2

Appendix S2. Outcrop area and sampled diversity of Karoo Basin therocephalians and cynodonts.

Karoo Biozone	Base (Ma)	Top (Ma)	Duration (Myr)	Outcrop area (total) (km^2)	Outcrop area (productive) (km^2)	#theroceph genera	# cyno genera
Cynognathus AZ	248	241.2	6.8	11,196	5,488	2	8
Lystrosaurus AZ	252.3	248	4.3	76,550	48,358	7	4
Dicynodon AZ	255	252.3	2.7	41,113	20,322	11	3
Cistecephalus AZ	257	255	2.0	41,082	31,360	8	2
Tropidostoma AZ	259.5	257	2.5	17,718	14,739	6	2
Pristerognathus AZ	262	259.5	2.5	3,857	3,857	5	--
Tapinocephalus AZ	265.8	262	3.8	21,043	15,806	7	--
Eodicynodon AZ	268	265.8	2.2	1,286	1,066	2	--

Ma, millions of years ago (mega-annum); Myr, million years; dates are estimates based on Abdala and Ribeiro (2010), Smith et al. (2012), and Rubidge et al. (2013); AZ, Assemblage Zone;
Outcrop area estimates from King (1991);
References: Abdala F, Ribeiro AM (2010) Distribution and diversity patterns of Triassic cynodonts (Therapsida, Cynodontia) in Gondwana. Palaeogeogr Palaeoclimatol Palaeoecol 286:202–217; Smith RMH, Rubidge BS, van der Walt M (2012) Therapsid biodiversity patterns and paleoenvironments of the Karoo Basin, South Africa. In: Chinsamy-Turan A, editor. Forerunners of Mammals: Radiation, Histology, Biology. Bloomington: Indiana University Press. pp. 31–62; Rubidge BS, Erwin DH, Ramezani J, Bowring SA, de Klerk WJ (2013) High-precision temporal calibration of Late Permian biostratigraphy: U-Pb constraints from the Karoo Supergroup, South Africa. Geology 41:363–366; King G (1991). Terrestrial tetrapods and the end Permian event: a comparison of analyses. Hist Biol 5:239–255.
