# Supplementary material for: Body Size Reductions in Nonmammalian Eutheriodont Therapsids (Synapsida) during the End-Permian Mass Extinction
Source: PLoS One. 2014 Feb 3;9(2):e87553. doi: 10.1371/journal.pone.0087553 (PMC3911975; doi:10.1371/journal.pone.0087553)
Supplement: Appendix S3 — Age and clade rank data for therocephalian and cynodont subclades. Basal skull length measurements (BSL) are in mm. (PDF) [file pone.0087553.s003.pdf]

Appendix S3

Appendix S3. Age and clade rank data for therocephalian and cynodont subclades. Basal skull length measurements (BSL) are in mm.

Eutheriodontia	**CR**†	**AR**‡	**MaxBSL**	**lnBSL**	**Voucher specimen/Source**
Therocephalia					
Lycosuchus (CR1)	1	2	340	5.828	SAM-PK-9084
Scylacosauridae (CR2)					
Ictidosaurus	2	1	168*	5.123	Abdala et al. [72] (NMQR 2910)
Glanosuchus	2	2	330	5.799	UCMP 42669
Pristerognathus	2	2	262	5.568	SAM-PK-11492
Scylacosuchus (CR3)	3	3	250*	5.521	Ivakhnenko [73] (PIN 2628/1)
Akidnognathidae (CR4)					
Annatherapsidus	4	5	250*	5.521	Ivakhnenko [73] (PIN 2005/1993)
Akidnognathidae, n. sp.	4	5	218	5.384	USNM 412421
Akidnognathus	4	5	150	5.010	BP/1/499
Promoschorhynchus	4	6	125	4.828	RC 116
Olivierosuchus	4	7	122	4.804	NMQR 3605
Euchambersia	4	5	115	4.744	NHMUK R5696
Cerdosuchoides	4	6	134	4.897	RC 58
Moschorhinus	4	6	260	5.560	BP/1/3983
Unnamed clade (CR5)					
Hofmeyriidae, new sp.	5	5	60	4.094	SAM-PK-K8516
Hofmeyria	5	3	89	4.488	SAM-PK-10525
Mirotenthes	5	5	110	4.700	UCMP 40467
Ictidostoma	5	3	74	4.304	CGS CM86-258
Ictidochampsa	5	6	115	4.744	RC 69
Viatkosuchus	5	4	170	5.135	Tatarinov [74] (PIN 2212/13)
Moschowhaitsia	5	6	250*	5.521	Ivakhnenko [73] (PIN 1100/20)
Theriognathus	5	5	336	5.817	BP/1/4008
Ictidosuchidae (CR6)					
Ictidosuchus	6	4	150	5.010	SAM-PK-K10650
Ictidosuchoides	6	3	146	4.983	SAM-PK-K6886
Regisaurus (CR7)	7	7	123	4.812	CGS AK86/7
Unnamed clade (CR8)					
Karenites	8	4	100*	4.605	Ivakhnenko [73] (PIN 2212/93)
Zambian karenitid	8	5	77	4.343	NHCC LB44
Lycideops	8	6	210	5.347	NHMUK R5695
Choerosaurus	8	4	89	4.488	SAM-PK-8797
Tetracynodon tenuis	8	6	63	4.143	RC 89
Tetracynodon darti	8	7	75	4.317	UCMP 42869
Scaloposaurus (CR9)	9	7	67	4.204	NMQR 3323
Ericiolacertidae (CR10)					
Ericiolacerta	10	7	59	4.077	BP/1/4794
Silphedosuchus	10	7	35*	3.555	Ivakhnenko [73] (PIN 951/100)
Ordosiidae (CR11)					
Ordosiodon	11	9	101	4.615	Hou [75] (IVPP V4786)
Hazhenia	11	8	157	5.056	Sun and Hou [76] (IVPP V5866)
Bauriidae (CR12)					
Bauria	12	9	130	4.867	AMNH FR 5622
Microgomphodon	12	8	87	4.465	SAM-PK-K10160

	CR	AR	MaxBSL	lnBSL	Voucher specimen/Source
Cynodontia					
Basal cynodont, n. sp.	n/a	6	185	5.220	SAM-PK-K10702
Charassognathus (CR1)	1	4	63	4.143	SAM-PK-K10369
Dvinia (CR2)	2	6	88	4.477	PIN 2005/2469
Procynosuchus (CR3)	3	4	60	4.094	SAM-PK-K10138
Galesauridae (CR4)					
Cynosaurus	4	5	48	3.871	BP/1/1563
Galesaurus	4	7	120	4.787	NMQR 860
Progalesaurus	4	7	93	4.532	SAM-PK-K9954
Thrinaxodon (CR5)	5	7	100	4.605	SAM-PK-K1461
Platycraniellus (CR6)	6	7	84	4.430	Abdala [77] (TM 25)
Cynognathia (CR7)					
Cynognathus	7	8	330	5.799	SAM-PK-11484
Diademodon	7	9	265	5.579	BP/1/3754
Sinognathus	7	9	121	4.795	Abdala et al. [78] (IVPP V2339)
Beishanodon	7	8	232*	5.446	Gao et al. [79] (PKUP V3007)
Trirachodon	7	8	105	4.653	SAM-PK-K10762
Langbergia	7	8	115	4.744	BP/1/5362
Cricodon	7	9	160	5.075	Abdala et al. [78] (BP/1/5540)
Luangwa	7	9	142	4.955	BP/1/3731
Dadadon	7	10	143	4.962	Ranivoharimanana et al. [60] (FMNH PR2232)
Massetognathus	7	10	204	5.318	Abdala and Giannini [80] (PULR11)
Protuberum	7	10	200	5.298	Reichel et al. [81] (MGB 368-100)
Exaeretodon	7	11	400	5.991	Abdala et al. [82] (MCZ 4486)
Lumkuia (CR8)	8	9	58	4.060	BP/1/2669
Unnamed clade (CR9)					
Ecteninion	9	11	98	4.584	Martinez et al. [83] (PVSJ 422)
Trucidocynodon	9	11	187	5.231	Oliveira et al. [58] (UFRGS PV1051T)
Chiniquodontidae (CR10)					
Chiniquodon	10	10	310	5.736	Abdala and Giannini [84] (UFRGS PV0122T)
Aleodon	10	9	156	5.049	Abdala and Smith [85] (GSN EN3)
Probainognathus (CR11)	11	10	83	4.418	Romer [86]; Sidor [52] (MCZ 4277)
Therioherpeton (CR12)	12	11	33*	3.496	Oliveira [87] (MVP05.22.04)
Ictidosauria (CR13)					
Riograndia	13	12	35	3.555	Bonaparte et al. [88]; Soares et al. [89] (MCN PV2264; UFRGS PV0569T)
Chaliminia	13	13	48	3.871	Martinelli and Rougier [90] (PVL 3257)
Elliotherium	13	13	58	4.060	BP/1/6106
Brasilodontidae (CR14)					
Brasilodon	14	12	26	3.258	Bonaparte et al. [91] (UFRGS PV0628T)
Brasilitherium	14	12	27	3.295	Bonaparte et al. [92] (UFRGS PV0929T)
Mammaliaformes (CR15)					
Sinoconodon	15	13	37	3.610	Patterson and Olson [93]; Crompton and Sun [94] (IVPP 4727; IVPP 8683; IVPP 8692)
Morganucodon	15	13	26*	3.258	Kermack et al. [95]

CR, clade rank; AR, age rank;
Asterisk (*) denotes estimated measurement from literature source;
† CR values correspond to side branches designated in Figure 3;

‡ AR values correspond to: 1, Wordian (Middle Permian) *Eodicynodon* AZ; 2, Capitanian (Middle Permian) *Tapinocephalus* AZ; 3, Capitanian-Wuchiapingian (Middle-Late Permian) *Pristerognathus* AZ; 4, Wuchiapingian (early Late Permian) *Tropidostoma* AZ; 5, Wuchiapingian (Late Permian) *Cistecephalus* AZ; 6, Wuchiapingian-Changxingian (Late Permian) *Dicynodon* AZ; 7, Induan-Olenekian (Early Triassic) *Lystrosaurus* AZ; 8, Olenekian (late Early Triassic) *Cynognathus* subzone A; 9, Anisian (early Middle Triassic) *Cynognathus* subzones B-C; 10, Ladinian (late Middle Triassic); 11, Carnian (early Late Triassic); 12, Norian (Late Triassic); 13, Rhaetian (latest Triassic).

Supplemental References

72. Abdala F, Rubidge BS, van den Heever J (2008) The oldest therocephalians (Therapsida, Eutheriodontia) and the early diversification of Therapsida. Palaeontology 51:1011–1024.

73. Ivakhnenko MF (2011) Permian and Triassic Therocephals (Eutherapsida) of Eastern Europe. Paleontological Journal 45:981–1144.

74. Tatarinov LP (1995) *Viatkosuchus sumini*, a new Late Permian therocephalian from the Kirov region. Paleontological Journal 29:111–128.

75. Hou L (1979) On a new theriodont from Inner Mongolia. Vertebrata Palasiatica 17:121-130.

76. Sun A, Hou L (1981) *Hazhenia*, a new genus of Scaloposauria. Acta Palaeontologica Sinica 20:297–311.

77. Abdala F (2007) Redescription of *Platycraniellus elegans* (Therapsida, Cynodontia) from the Lower Triassic of South Africa, and the cladistic relationships of eutheriodonts. Palaeontology 50:591–618.

78. Abdala F, Hancox PJ, Neveling J (2005) Cynodonts from the uppermost Burgersdorp Formation, South Africa, and their bearing on the biostratigraphy and correlation of the Triassic *Cynognathus* Assemblage Zone. J Vertebr Paleontol 25:192–199.

79. Gao K, Fox RC, Zhou CF, Li DQ (2010) A new nonmammalian eucynodont (Synapsida, Therapsida) from the Triassic of northern Gansu Province, China, and its biostratigraphic and biogeographic implications. American Museum Novitates 3685:24 pp.

80. Abdala F, Giannini NP. 2000. Gomphodont cynodonts of the Chañares Formation: the analysis of an ontogenetic sequence. J Vertebr Paleontol 20:501–506.

81. Reichel M, Schultz CL, Soares MB (2009) A new traversodontid cynodont (Therapsida, Eucynodontia) from the Middle Triassic Santa Maria Formation of Rio Grande do Sul, Brazil. Palaeontology 52:229–250.

82. Abdala F, Barberena MC, Dornelles J (2002) A new species of the traversodontid cynodont *Exaeretodon* from the Santa Maria Formation (Middle/Late Triassic) of southern Brazil. J Vertebr Paleontol 22:313–325.

83. Martinez RN, May CL, Forster CA (1996) A new carnivorous cynodont from the Ischigualasto Formation (Late Triassic, Argentina), with comments on eucynodont phylogeny. J Vertebr Paleontol 16:271–284.

84. Abdala F, Giannini NP (2002) Chiniquodontid cynodonts: systematic and morphometric considerations. Palaeontology 45:1151–1170.

85. Abdala F, Smith RMH (2009) A Middle Triassic cynodont fauna from Namibia and its implications for the biogeography of Gondwana. J Vertebr Paleontol 29:837–851.

86. Romer AS (1970) The Chañares (Argentina) Triassic reptile fauna. VI. A cynodont with an incipient squamosal-dentary articulation. Breviora 344:1–18.

87. Oliveira EV (2006) Reevaluation of *Therioherpeton cargnini* Bonaparte & Barberena, 1975 (Probainognathia, Therioherpetidae) from the Upper Triassic of Brazil. Geodiversitas 28:447–465.

88. Bonaparte JF, Ferigolo J, Ribeiro AM (2001) A primitive Late Triassic 'ictidosaur' from Rio Grande do Sul, Brazil. Palaeontology 44:623–635.

89. Soares MB, Schultz CL, Horn BL (2011) New information on *Riograndia guaibensis* Bonaparte, Ferigolo & Ribeiro, 2001 (Eucynodontia, Tritheledontidae) from the Late Triassic of southern Brazil: anatomical and biostratigraphic implications. Anais da Academia Brasileira de Ciências 83:329–354.

90. Martinelli AG, Rougier GW (2007) On *Chaliminia musteloides* (Eucynodontia: Tritheledontidae) from the Late Triassic of Argentina, and a phylogeny of Ictidosauria. J Vertebr Paleontol 27:442–460.

91. Bonaparte JF, Martinelli AG, Schultz CL, Rubert R (2003) The sister group of mammals: small cynodonts from the Late Triassic of southern Brazil. Revista Brasileira de Paleontología 5:5-27.

92. Bonaparte JF, Martinelli AG, Schultz CL (2005) New information on *Brasilodon* and *Brasilitherium* (Cynodontia, Probainognathia) from the Late Triassic of southern Brazil. Revista Brasileira de Paleontologia 8:25–46.

93. Patterson B, Olson EC (1961) A triconodont mammal from the Triassic of Yunnan. International Colloquium on the Evolution of Lower and Non Specialized Mammals. Koninklijke Vlaamse Academir voor Wetenschappen, Letteren en Schone Kunsten can Belgie 129–191.

94. Crompton AW, Sun AL (1985) Cranial structure and relationships of the Liassic mammal *Sinoconodon*. Zool J Linn Soc 85:99–119.

95. Kermack KA, Mussett F, Rigney HW (1981) The skull of *Morganucodon*. Zool J Linn Soc 71:1–158.
