# Supplementary material for: Body Size Reductions in Nonmammalian Eutheriodont Therapsids (Synapsida) during the End-Permian Mass Extinction
Source: PLoS One. 2014 Feb 3;9(2):e87553. doi: 10.1371/journal.pone.0087553 (PMC3911975; doi:10.1371/journal.pone.0087553)
Supplement: Dataset S1 — Phylogenetic methods and supporting NEXUS file. (PDF) [file pone.0087553.s004.pdf]

Supporting Dataset S1

Phylogeny. An informal supertree was constructed from several literature sources, including: therocephalians, Huttenlocker [54], Huttenlocker et al. [33], Sigurdsen et al. [55]; cynodonts, Hopson & Kitching [56], Abdala et al. [45], Sidor & Hancox [57], Ranivoharimanana et al. [60], Oliveira et al. [58], Liu & Olsen [59]. Additional therocephalian taxa were included for completeness and a comprehensive reanalysis of their cladistic relationships was performed and appended to the supertree, updated from Huttenlocker [54], Huttenlocker et al. [33], and Sigurdsen et al. [55]. A matrix was assembled containing 135 craniodental and postcranial characters, and 56 taxa (54 ingroup taxa, including 49 therocephalians) with two outgroups (*Biarmosuchus* and *Titanophoneus*). Character lists were arranged into a set of individual "character statements" composed of an anatomical locator, variable and/or variable qualifier, and their respective character states. This formula was preferred to minimize ambiguity in homology statements and improve data comparison. Six additional characters (chars. 130–135) and 22 new ingroup taxa were added to the matrix of Sigurdsen et al. [55]. Newly coded taxa included: *Alopecodon priscus* (SAM-PK-920; AMNH 5569), *Pardosuchus whaitsi* (SAM-PK-1077), *Scylacosaurus sclateri* (SAM-PK-634; SAM-PK-11888), *Scylacosuchus orenburgensis* (Ivakhnenko [73]; Tatarinov [99]), *Perplexisaurus foveatus* (Ivakhnenko [73]; Tatarinov [100]), *Chthonosaurus velocidens* (PIN 521/1), a new Zambian chthonosaurid (NHCC LB36, 38), *Annatherapsidus petri* (Ivakhnenko [73]; Tatarinov [99]), a new *Cistecephalus* AZ akidnognathid (USNM PAL412421), *Akidnognathus parvus* (SAM-PK-4021; BP/1/499; BP/1/641), a new *Cistecephalus* AZ hofmeyriid (SAM-PK-K8516), *Ictidochampsa platyceps* (RC 69), a new *Tropidostoma* AZ whaitsiid (SAM-PK-K10990, K10984), *Urumchia lii* (Young [101]; Sun [102]), *Karenites ornamentatus* (Tatarinov [103, 104]; Ivakhnenko [73]), a new Zambian karenitid (NHCC LB44), *Silphedosuchus orenburgensis* (Ivakhnekno [73]; Battail and Surkov [105]), *Nothogomphodon danilovi* (Ivakhnenko [73]; Tatarinov [99]), *Hazhenia concava* (Sun and Hou [76]; Sun [102]), *Ordosiodon youngi* (Hou [75]; Sun [102]), *Antecosuchus ochevi* (Ivakhnenko [73]; Tatarinov [99]), and *Traversodontoides wangwuensis* (Sun [106]; Sun [102]).

The data were analyzed using the maximum parsimony criterion of PAUP 4.0 beta 10 [107], and a Bayesian analysis in MrBayes v. 3.1.2 [108]. In PAUP, I performed the analysis using the tree-bisection-reconnection branch-swapping algorithm with DELTRAN optimization. Initially, all characters were unordered and had equal weight. I then ran an ordered trial using the same parameters, but ordering characters 40, 43, and 57. In both unordered and ordered trials, multistate taxa were treated as polymorphisms. A heuristic search was performed using a random addition sequence with 100 replicates. A bootstrap analysis was also performed as an additional measure of clade support, using 1000 bootstrap replicates. The Bayesian analysis was performed using flat prior probabilities and standard settings (*Mk* model for morphological evolution; Lewis [109]), running the analysis until the standard deviation of split frequencies fell below 0.01 (one million generations) and sampled every 100 generations. Analyses were ran with the gamma distribution parameter allowing variable character rates. A consensus topology was manually built in Mesquite [62] and the tree was pruned to include only taxa with complete skull length data (Fig. S1) and appended to the cynodont reference cladogram (Fig. 2).

References:

99. Tatarinov LP (1974) Theriodonts of the USSR. Trudy Paleontologicheskogo Instituta, Akademii Nauk SSSR 143:1–240. [in Russian]

100. Tatarinov LP (1997) A new Scaloposaurid (Reptilia, Theriodontia) with an extraordinary sensory system from the Upper Permian of the Kirov Region. Paleontological Journal 31:669–676.

101. Young C-c (1952) On a new therocephalian from Sinkiang, China. Acta Scientia Sinica 1:152–165.

102. Sun A (1991) A review of Chinese therocephalian reptiles. Vertebrata Palasiatica 29:85–94.

103. Tatarinov LP (1995) A new ictidosuchid *Karenites ornamentatus* (Theriodontia) from the Upper Permian of the Kotel'nich Locality in the Kirov Region. Russian Journal of Herpetology 2:18–33.

104. Tatarinov LP (1999) The first Scaloposaurid (Reptilia, Theridontia) from Russia (Upper Permian, Kirov Region). Paleontological Journal 33:278–288.

105. Battail B & Surkov MV (2000) Mammal-like reptiles from Russia. The Age of Dinosaurs in Russia and Mongolia, eds Benton MJ, Shishkin MA, Unwin DM, & Kurochkin EN (Cambridge University Press, Cambridge), pp. 86–119.

106. Sun A (1981) Reidentification of *Traversodontoides wangwuensis* Young. Vertebrata Palasiatica 19:1–4.

107. Swofford D (1999) PAUP* phylogenetic analysis using parsimony, Version 4.0 beta 10. Sinauer Associates, Sunderland, MA.

108. Ronquist F, van der Mark P, & Huelsenbeck JP (2009) Bayesian phylogenetic analysis using MrBayes. The Phylogenetic Handbook 2nd Edition, eds Vandamme AM, Salemi M, & Lemey P (Cambridge University Press, Cambridge), pp. 210–266.

109. Lewis PO (2001) A likelihood approach to estimating phylogeny from discrete morphological character data. Systematic Biology 50:913–925.

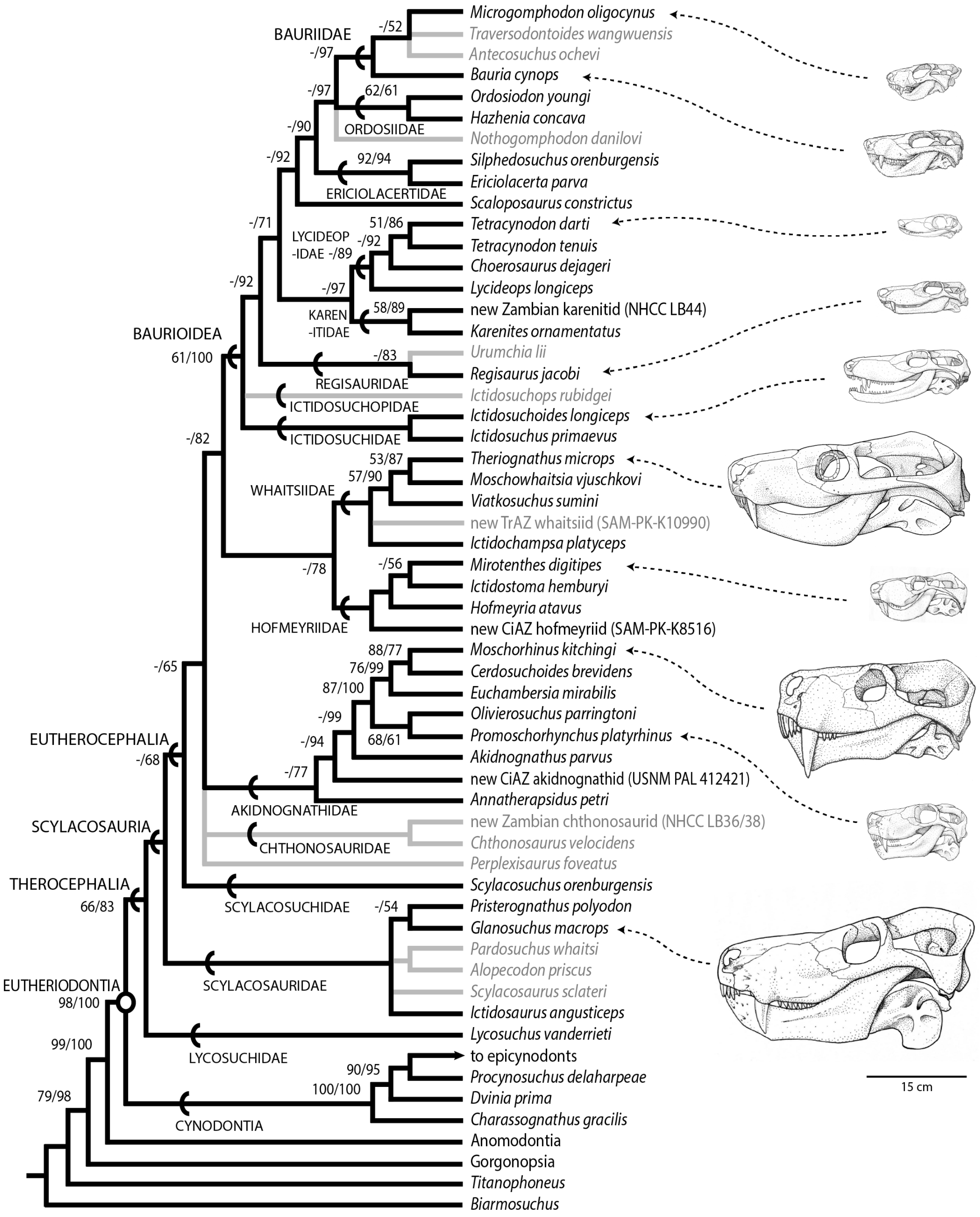

Figure S1. Hypothesis of the cladistic relationships of therocephalian eutheriodonts based on maximum parsimony and Bayesian inference (tree length, 412 steps; retention index, 0.788; consistency index, 0.487). Taxa/branches in light gray were excluded from the composite supertree (Fig. 2) as skull length data were unavailable for those taxa. Numbers at nodes indicate node support: bootstrap values ≥ 50%, left of virgule; Bayesian posterior probabilities, right of virgule. (Based on Huttenlocker [67]: fig.1.9.)

NEXUS Script.

```
#NEXUS
[ File output by Morphobank v3.0 (http://www.morphobank.org); 2012-12-13 18.22.50 ]
BEGIN TAXA;
	DIMENSIONS NTAX=56;
	TAXLABELS
		'Biarmosuchus_tener'
		'Titanophoneus_potens'
		'Gorgonopsia'
		'Anomodontia'
		'Charassognathus_gracilis'
		'Dvinia_prima'
		'Procynosuchus_delaharpeae'
		'Lycosuchus_vanderrieti'
		'Alopecodon'
		'Glanosuchus_macrops'
		'Ictidosaurus_angusticeps'
		'Pardosuchus'
		'Pristerognathus_polyodon'
		'Scylacosaurus'
		'Scylacosuchus'
		'Annatherapsidus'
		'Cist_AZ_akidnog_USNM_PAL_412421'
		'Akidnognathus_parvus'
		'Euchambersia_mirabilis'
		'Cist_AZ_hofmeyriid_SAM-PK-K8516'
		'Promoschorhynchus'
		'Olivierosuchus_parringtoni'
		'Cerdosuchoides'
		'Moschorhinus_kitchingi'
		'Chthonosaurus'
		'Zambian_chthonosaurid_NHCC_LB36-38'
		'Ictidostoma_hemburyi'
		'Trop_AZ_whaitsiid_SAM-PK-K10990'
		'Hofmeyria_atavus'
		'Mirotenthes_digitipes'
		'Ictidochampsa_platyceps'
		'Viatkosuchus_sumini'
		'Moschowhaitsia_vjuschkovi'
		'Theriognathus_microps'
		'Ictidosuchus_primaevus'
		'Ictidosuchoides_longiceps'
		'Perplexisaurus'
		'Ictidosuchops_rubidgei'
		'Regisaurus_jacobi'
		'Urumchia'
		'Choerosaurus_dejageri'
```

```
                'Lycideops_longiceps'
                'Karenites_ornamentatus'
                'Zambian_karenitid_NHCC_LB44'
                'Tetracynodon_tenuis'
                'Tetracynodon_darti'
                'Scaloposaurus_constrictus'
                'Ericiolacerta_parva'
                'Silphedosuchus'
                'Nothogomphodon'
                'Ordosiodon'
                'Hazhenia'
                'Antecosuchus'
                'Bauria_cynops'
                'Traversodontoides'
                'Microgomphodon_oligocynus'
        ;
ENDBLOCK;



BEGIN CHARACTERS;
        DIMENSIONS NCHAR=135;
        FORMAT DATATYPE=STANDARD MISSING=? GAP=- SYMBOLS="0123456789";

        CHARLABELS
                [1] 'Premaxilla dorsal process'
                [2] 'Septomaxilla shape'
                [3] 'External nares '
                [4] 'Dorsal profile of antorbital region '
                [5] 'Constriction of snout directly behind caniniforms'
                [6] 'Preorbital depression anterior to a thickened ridge on anterior margin of orbit'
                [7] 'Maxillary facial plate'
                [8] 'Concave ventral step in maxillary facial plate between caniniforms or anterior most maxillary teeth and incisors'
                [9] 'Broad excavation or pit in maxilla immediately posterior to dominant canine'
                [10] 'Posterior region of maxillary facial plate is folded inward onto palatal region so that maxilla is well exposed in ventral view just anterior to orbit'
                [11] 'Suborbital bar robusticity'
                [12] 'Suborbital bar lateral expansion'
                [13] 'Rostrum height relative to orbit position'
                [14] 'Anterior border of orbit located on'
                [15] 'Jugal postorbital process'
                [16] 'Postorbital bar robusticity'
                [17] 'Zygomatic arch orientation behind orbit'
                [18] 'V shaped posterior border of nasals pointing toward the occiput'
                [19] 'Median fronto nasal crest'
```

[20] 'Postfrontal'
[21] 'Parietal pineal opening in adults'
[22] 'Temporal fenestra relative to orbit size in adults'
[23] 'Zygomatic arch robusticity'
[24] 'Participation of parietal in temporal fenestra dorsal border'
[25] 'Parietal expanded posteriorly on midline behind region of parietal or pineal housing'
[26] 'Parietal sagittal crest length if applicable'
[27] 'Posterodorsal inclination of the temporal region reaching its maximum height where the parietal crest meets the lambdoidal occipital crest if applicable'
[28] 'Intertemporal width in adults'
[29] 'Anteroposterior enlargement of the temporal fenestra'
[30] 'Posteroventral process of squamosal'
[31] 'Medially directed process of squamosal contacting prootic'
[32] 'Parietal crest anterior position'
[33] 'Nasal lacrimal contact'
[34] 'Prefrontal postorbital contact in adults'
[35] 'Greatest width of zygomatic arches'
[36] 'Upturning of alveolar margin of premaxilla'
[37] 'Interorbital width'
[38] 'Jugal anterior extent'
[39] 'Palatal fenestra for lower caniniform'
[40] 'Palatal processes of the maxillae'
[41] 'Vomer premaxilla process shape'
[42] 'Vomer premaxilla contact'
[43] 'Vomer fusion'
[44] 'Ventromedian crest between palatines on posterior portion of vomer'
[45] 'Palatine teeth'
[46] 'Large suborbital vacuities bound by the palatine pterygoid and ectopterygoid'
[47] 'Pterygoid ventromedian tubercle crest'
[48] 'Pterygoid transverse flange teeth'
[49] 'Pterygoid boss teeth'
[50] 'Pterygoid transverse flange lateral expansion'
[51] 'Pterygoid transverse flange position'
[52] 'Palatal exposure of maxilla behind canine'
[53] 'Formation of secondary palate and incisive fissure if applicable'
[54] 'Parasagittal ridges running from medial posterior flare of transverse flanges to basioccipital'
[55] 'Vomer anterior vault'
[56] 'Epipterygoid parietal contact'
[57] 'Epipterygoid ascending process'
[58] 'Posterior apophysis of the epipterygoid contacting or nearly contacting the prootic'
[59] 'Laterally directed processes of the prootic participating in the pterygoparoccipital foramen'
[60] 'Basal tubera'
[61] 'Dorsal surface of the paroccipital process'

[62] 'Paroccipital process of the opisthotic orientation'
[63] 'Opisthotic dorsolateral process'
[64] 'Mastoid process'
[65] 'Tabular-paroccipital process of opisthotic contact'
[66] 'Tabular ventral extent'
[67] 'Trigeminal foramen'
[68] 'Epipterygoid alisphenoid prootic overlap'
[69] 'Epipterygoid frontal contact'
[70] 'Occipital condyle'
[71] 'Tabular dorsal contribution to lambdoidal crest'
[72] 'Quadrate and quadratojugal size'
[73] 'Quadrate and quadratojugal situated in a depression on the anterior face of the squamosal quadrate recess'
[74] 'Dorsal process of stapes'
[75] 'Posteroventral process on quadrate in posterior notch quadrate recess of squamosal'
[76] 'Stapedial foramen structure and orientation'
[77] 'Overall dentary shape'
[78] 'Dentary anterior shape and mentum'
[79] 'Specialized boss on posteroventral portion of dentary ramus'
[80] 'Mandibular symphysis in ventral view'
[81] 'Lateral surface of dentary in ventral view'
[82] 'Dentary angle lateral to the reflected lamina'
[83] 'Coronoid process posterodorsal terminal margin shape if applicable'
[84] 'Coronoid process dorsal extent in adults if applicable'
[85] 'Dentary height increased posteriorly and postdentary bones reduced to form a free standing coronoid process'
[86] 'Postdentary bones height relative to total dentary height'
[87] 'Ventral margins of angular and dentary'
[88] 'Splenial lateral exposure'
[89] 'Mandibular fenestra'
[90] 'Reflected lamina shape and ventral extent'
[91] 'Dentary masseteric fossa in adults'
[92] 'Reflected lamina of angular tympanic size'
[93] 'Space between left and right dentaries '
[94] 'Lateral sulcus along dentary ramus and coronoid process'
[95] 'Interlocking incisors'
[96] 'Upper incisor count'
[97] 'Upper incisor surface texture'
[98] 'Incisor shape'
[99] 'Functional upper precanines in adults if applicable'
[100] 'Upper dominant canine maxillary in adults if applicable'
[101] 'Deep pit or groove running vertically along the anterior surface of the dominant canine'
[102] 'Upper postcanine number'
[103] 'Lower canine in adults if applicable'
[104] 'Lower postcanine teeth'
[105] 'Incisor cutting margins'

[106] 'Postcanine shape if applicable'
[107] 'Lower incisor count'
[108] 'Relative length of maxillary tooth row if applicable'
[109] 'Caudal vertebrae count'
[110] 'Scapular blade shape'
[111] 'Procoracoid foramen'
[112] 'Ossified cleithrum'
[113] 'Interclavicle shape'
[114] 'Humerus shape'
[115] 'Ectepicondylar foramen of humerus'
[116] 'Manual digit III shape of second phalanx'
[117] 'Manual digit IV phalangeal number'
[118] 'Sharp anterior process of dorsal iliac plate'
[119] 'Pubis and ischium orientation'
[120] 'Obturator foramen size'
[121] 'Obturator foramen position'
[122] 'Distinct trochanter minor on femur'
[123] 'Clavicles'
[124] 'Humeral head'
[125] 'Radius width relative to ulna'
[126] 'Femoral head'
[127] 'Calcaneal tuber tuber calcis'
[128] 'Premaxillary foramina on palate'
[129] 'Angle of incidence between adjoining maxillary processes on surface of secondary palate'
[130] 'Parietal crest fusion at midline'
[131] 'Maxillary postcanine alveolar margin in ventral view'
[132] 'Upper postcanine diastema immediately behind dominant caniniform'
[133] 'Lacrimal foramina or pits exposed ventrolaterally outside orbital wall in a separate anteorbital fossa'
[134] 'Suborbital vacuity shape'
[135] 'Premaxilla vomerine process contacts maxilla on medial border of lower canine fossa'
;

STATELABELS
1
'blunt and does not overhang incisors'
'pointy, forming a rostral process which projects over recumbent upper incisors'
,
2
'long, narrow element, moderately well exposed on the facial region '
'enlarged and well exposed outside of the external naris, broadly overlapping the premaxilla anteriorly'
,
3
'moderately large and face anterolaterally '
'enlarged close-set and face more anteriorly '

,
4
'convex'
'relatively straight'
,
5
'absent'
'present, producing bowling pin-shaped rostrum'
,
6
'absent'
'present extending anteroventrally from lacrimal to canine buttress of maxilla '
,
7
'high'
'low with a height less than 40% its length'
,
8
'present'
'absent'
,
9
'absent'
'present'
,
10
'absent'
'present'
,
11
'shallow'
'deep'
,
12
'no lateral expansion'
'slight expansion, extending from jugal onto posterior part of maxillary facial plate, and contributing to a pronounced degree of orbital convergence (i.e., orbits facing forward and appearing more triangular than oval in dorsal view)'
,
13
'rostrum high and narrow'
'rostrum long, low anterior to orbit'
,
14
'posterior half of the skull'
'anterior half of skull'
'transverse midline'
,

15
'present'
'absent'
,
16
'moderately well-built'
'extremely slender'
'absent unossified'
,
17
'posteroventral orientation'
'relatively straight or horizontal'
'curved having a concave ventral margin throughout its length'
,
18
'absent'
'present'
,
19
'absent'
'present'
,
20
'present'
'absent'
,
21
'present irrespective of ontogenetic stage'
'absent extremely reduced'
,
22
'subequal in size'
'larger than orbit'
,
23
'moderately deep'
'slender'
,
24
'absent'
'present'
,
25
'absent'
'present'
,
26
'moderate just over half the temporal fenestra length'

'short less than half the temporal fenestra length'
,
27
'present'
'absent'
,
28
'wide'
'narrow with vertical lateral faces'
,
29
'absent'
'present'
,
30
'absent'
'present'
,
31
'absent'
'present enclosing the pterygoparoccipital foramen'
,
32
'located posteriorly'
'extends forward in adults to include the pineal enclosure'
,
33
'absent'
'present'
,
34
'absent'
'present only on orbit dorsomedial wall'
'present dorsally excluding frontal from orbit margin'
,
35
'toward middle of arch'
'at back of arch, forming triangular skull outline in dorsal view'
,
36
'moderate to pronounced'
'absent or horizontal'
,
37
'wide approximately 20% of total skull length or greater'
'narrow significantly less than 20% total skull length'
,
38

'extends anteriorly beyond the anterior margin of the orbit'
'restricted to anterior margin of orbit'
,
39
'absent'
'present and confluent with internal naris '
'a separate anterior choanal housing for the lower canine is created by an extension of the premaxilla and maxilla from the primary palate'
'a fossa for the lower canine is present where the maxilla and premaxilla meet on the ventral surface of the secondary palate'
,
40
'absent'
'form a well developed crista choanalis with a ridge extending posteriorly onto the palatine'
'contact or nearly contact the ventrally extending vomer at a shallow angle with no sutural connection'
'contact vomer with a strong but short sutural connection'
'contact the vomer at a strong angle creating a concave anteromedial surface on the crista choanalis, and bearing a moderately long sutural connection with the lateral margins of the vomer'
'meet at the midline, sharing a sutural connection and obscuring most of the vomer on the palatal surface '
'small anteriorly located processes meet specialized lateral processes of vomer, dividing choana into anterior and poster regions'
,
41
'slightly bulbous between choanae, narrowing toward its contact with the premaxilla'
'expands anteriorly and is widest at its contact with the premaxilla'
'bears specialized transverse processes just behind contact with premaxilla contacting vomerine processes of crista choanalis'
,
42
'anteriormost portion of vomer briefly contacts vomerine process of premaxilla'
'broadly overlaps the ventral surface of the premaxilla vomerine process'
,
43
'paired'
'fused anteriorly'
'completely fused'
,
44
'absent'
'present'
,
45
'present'

'absent'
,
46
'absent or reduced in adults'
'present throughout ontogeny'
,
47
'absent'
'present anterior to pterygoid vacuity'
,
48
'present'
'absent'
,
49
'present'
'absent'
,
50
'moderate'
'reduced'
'sharp, posteriorly projecting wings with slight posterolateral expansion'
,
51
'more anterior to the center of the orbit'
'more posterior to the center of the orbit'
,
52
'less than 20% distance from canine to posterior end of palatine'
'greater than 20% distance from canine to posterior end of palatine'
,
53
'formation of secondary palate occurs such that the posterior portion of the maxillae and palatines approach at the midline but are slightly open anteriorly thus creating an incipient incisive fissure or foramen'
'anterior portion is more closed than the posterior leaving no indication of an incisive foramen or fissure'
,
54
'absent'
'present'
,
55
'present'
'absent'
,
56
'epipterygoid separate from parietal'

'epipterygoid contacts parietal'
,
57
'appears as a thin rod'
'is slightly expanded anteroposteriorly'
'is extremely expanded, the greatest dorsal anteroposterior length being almost equal to the dorsal height '
,
58
'absent'
'present, enclosing an aperture presumably for the trigeminal nerve'
,
59
'absent'
'present'
,
60
'small to moderate in size'
'large one-third occipital breadth'
,
61
'relatively smooth or straight'
'deeply hollowed in the floor of the posttemporal fenestra'
,
62
'strongly posteroventral relative to horizontal'
'moderately posteroventral relative to horizontal'
'transverse'
,
63
'absent'
'present and contacts tabular and/or squamosal, thus excluding supraoccipital from contact with post-temporal fenestra'
,
64
'absent or poorly developed'
'squamosal and paroccipital process of the opisthotic form a distinct posteriorly projecting mastoid process'
,
65
'tabular contacts paroccipital process'
'withdrawn from contact with paroccipital process'
,
66
'extends below the post-temporal fenestra'
'just barely contacts the post-temporal fenestra'
,
67

'Trigeminal nerve exit situated between prootic incisure and epipterygoid'
'Trigeminal nerve exits via a specialized foramen formed between the prootic and epipterygoid'
,
68
'absent'
'present'
,
69
'absent'
'present'
,
70
'single'
'double'
,
71
'low and broad'
'high nearly contacting dorsal margin of interparietal with significant contribution to lambdoidal crest'
,
72
'relatively large'
'reduced in height'
,
73
'absent'
'present'
,
74
'present'
'reduced or absent'
,
75
'absent'
'present'
,
76
'oriented posteroventrally'
'reduced or absent'
,
77
'deep/robust'
'short and banana-shaped'
'long, slender, and relatively straight with a smooth ventral edge'
,
78
'dentary deepens anteriorly'

'dentary continuously tapers to a narrow anterior edge'
,
79
'absent'
'present'
,
80
'only moderately expanded mediolaterally with a low mentum angulation in ventral view'
'anteroposteriorly thickened with strong suture'
,
81
'relatively smooth'
'bears a marked constriction behind the lower canine where upper canine rests'
,
82
'absent smoothly rounded'
'moderate sharp'
'pronounced protruding with an angle 120 degrees'
,
83
'straight'
'more rounded'
'comes to a sharp point'
,
84
'terminates below middle of orbit'
'terminates in upper half of orbit'
,
85
'absent'
'present'
,
86
'equal'
'between 1/2 and equal'
,
87
'confluent'
'angular (=tympanic) positioned dorsal to ventral margin of dentary'
,
88
'exposed laterally near symphysis'
'obscured by dentary'
,
89
'absent'
'present, penetrating the mandible and visible laterally'

'surangular above and prearticular below a small fenestra on the medial surface of the mandible'
,
90
'rounded, projecting below the ventral margin of the dentary at about the level of the second groove'
'slightly anteroposteriorly elongate spade shaped and does not appear to extend below the dentary'
'extremely reduced and spoon shaped'
,
91
'absent'
'present, positioned high on coronoid process'
,
92
'large'
'reduced'
,
93
'widens greatly posteriorly'
'remains relatively long and narrow just posterior to symphyseal region'
,
94
'absent'
'present'
,
95
'present'
'absent'
,
96
'few, up to five'
'six'
'seven or more'
,
97
'smooth'
'longitudinal fluting or faceting'
,
98
'relatively straight and conical'
'spatulate concave lingually with mesiolingual and distolingual crests'
,
99
'absent'
'present'
,
100

'large relative to maxilla height'
'medium'
'extremely reduced'
,
101
'absent'
'present'
,
102
'numerous, more than five'
'few, five or less'
'absent'
,
103
'large'
'medium'
'reduced or absent'
,
104
'present'
'absent'
,
105
'serrated'
'smoothly ridged'
,
106
'tall with simple, sharp apices lacking cusps'
'two or more cusps in line'
'short, fat cones'
,
107
'four'
'less than four'
'more than four'
,
108
'greater than 50% the total length of maxilla'
'approximately 50% or less than total length of maxilla'
,
109
'much more than 15'
'less than 15'
,
110
'short and broad'
'moderate breadth'
'delicate and narrow'

,
111
'bound within procoracoid'
'near or within procoracoid-coracoid suture'
'near or within procoracoid-scapula suture'
,
112
'present'
'absent'
,
113
'long and thin oblong or oval'
'short and broad'
'cruciform'
,
114
'short and robust'
'long and slender'
,
115
'present'
'absent'
,
116
'short disc-like but present'
'absent'
,
117
'5'
'4'
'3'
,
118
'absent'
'present above a ventral concavity'
,
119
'relatively vertical'
'more horizontal, forming a broad puboischiatic plate'
,
120
'small'
'moderately enlarged'
'extremely enlarged'
,
121
'situated entirely within pubis'
'bordered by pubis and ischium'

,
122
'absent'
'present'
,
123
'broad medially'
'narrow medially'
,
124
'convex articular surface extends broadly across head'
'articular surface bulbous and inflected'
,
125
'equal to or narrower distally'
'broader distally'
,
126
'elongate to subspherical'
'oblong and spherical'
,
127
'absent'
'present and oriented posteriorly'
,
128
'small or absent'
'large'
'single foramen medially'
,
129
'shallow'
'smoothly arching'
,
130
'forms narrow sagittal crest joined tightly behind a bulbous pineal housing'
'weak such that parasagittal contributions of left and right parietals are continuous with the temporal line and bound a narrow fissure'
,
131
'straight to slightly concave along buccal margin, bowing inward toward the midline'
'buccally convex at anterior extent, bowing outward away from the midline'
,
132
'present, greater than the spacing between successive postcanines'
'absent'
,

133
'absent'
'present'
,
134
'suboval about as long as wide'
'elongate and D-shaped to subtriangular with straight medial margins'
'broad shorter than wide due to anterior truncation by scalloped webbing'
,
135
'absent'
'present'
,
;

MATRIX
'Biarmosuchus_tener' 0000000000000000000000000--
0000?000000000??000000000-000?0000000000000000000000100--
000000000000000000000000000?00100000?00000??--000--
'Titanophoneus_potens' 00000000001000002?0001000--
0000?000010000000000000000-000000000000000000000000000000--
00000000000000000000000000000000012001?0?0000?--000--
'Gorgonopsia' 000{0,1}000000000000000001000--
0100?0000001000200000000-
01100000100000000011000000100201000000000?0000000000000?00000001000?0{0,1}00{0,
1}00--000--
'Anomodontia' 0000010?00000100200000100--
0110?000001?000001?00?11?-
00?0000010011000010100010001?02?1000{1,2}00000?000010010?010010?01012001?0111{0,1
}00--010?-
'Charassognathus_gracilis'
?0?1?000000101001001011110?1110?1200?1?????????????????120????????1101?11?1?2100
01001111?2?1?01?010100001110?????????????0??????00000??
'Dvinia_prima' 00011001000101001001111110011101120101 3-
00211001111100?1201?02?0001111?110102100010011 1?2211001101010000 11?0?????????0
02?0?????200010-0
'Procynosuchus_delaharpeae'
0001100100010100100101111001110112010 13-
002110011111100012 01?020000111111101021000100111022110011010100001100000??0001
002?011110000010-0
'Lycosuchus_vanderrieti'
00010000000100100210001011001110000100010100011101210-
10110?1??100?00000111?100000200111120000110000001000010?000?0????????11?1?0-
01000-
'Alopecodon'
?001?10100000?002??0?????????????00?1?0??????????????????????????????????????0000?2
??1111??0??111001000?00010???????????????????-?000?-

'Glanosuchus_macrops'
00011100000002002000010110011110001100111001111102101011101002?0110000?111110
0000100111120000112000000000010100?00112111??11{0,1}1?0-00000-

'Ictidosaurus_angusticeps'
000111000000020?2??0?111???11???00?1??111????????????0??????????????????????00000
200111120000112001000000010???????????????????-?000?-

'Pardosuchus'
?0011?0000000??????????????????????????1???????10???1??????????????????????000002
??1111??0?011100100???00?????????????????????-?000?-

'Pristerognathus_polyodon'
0001110000000200200001111001111000?10?111001111102101011101002?0110000?11???00
?001001111200001120000000000010??????????????????0-000?0-

'Scylacosaurus'
0001110000000?002000???1???1????00?10?11100011?102?0101??????????????????????????
??????????????2001000??00?0???????????????????-00000-

'Scylacosuchus'
0001010100??02???0?001?1100111?00011??11???????????01??11????21001??00??1?1?0000
01???????????11010100000102??1???01??????1???1??-0000?-

'Annatherapsidus'
?001000?00000200201?01111001111000110011102111110210111111?01210010000011?1?00
0?010011111?0001101010000010?0???????????????????0-01100-

'Cist_AZ_akidnog_USNM_PAL_412421'
?001?00100000200201101111001111000110021102111110210111????0121011??00111?????
?????????????????0101001??10?0???????????????????0-011000

'Akidnognathus_parvus'
111110010000020020110111100111100011002110211111121011111??0121????000111?1?00
01010011111?000110101000001000???????????????????0-01{0,1}000

'Euchambersia_mirabilis' 0111-
0011000010?20011111100111100211012111111011111011111?1012?0??0000?11??????????
???????000??0010012??1-?-???????????????????0-0--0-0

'Cist_AZ_hofmeyriid_SAM-PK-K8516'
???1000?0101010120010111110111?0011?011??????1?1?21011?????0????????????????1?0?
01111111??0?11???????000?0?0????????????????????-000000

'Promoschorhynchus'
111110010000020020110111110111110011012111111111121011111{0,1}101211110000111?
1?0001010011111000011011100{0,1}00100{0,1}?10111?????????1???0-011000

'Olivierosuchus_parringtoni'
11111001000002002011011110011111001{0,1}01211111111111210111111101211110000011?
??00010100111110000110{0,1}11001001001?10111112?????1111?0-011000

'Cerdosuchoides'
0111100000000{1,2}0020110111100111?100100121111111111210111????????????????11?1?
00011100111110000110010001001001?1?0???????????????-011000

'Moschorhinus_kitchingi'
0111100000000100201101111001111100100121111111111210111111101210110000111???0
0011100111110000110010001001001?????01????????111?0-011000

'Chthonosaurus'
???1?00?00000?002011011110?111?1001??0????211111021?11111??012????0000?11????1?
??????????????????????00??2?0????????????????????00?02?

'Zambian_chthonosaurid_NHCC_LB36-38'
???1?0??0?0?0?0??001?1?11??11?1?00???0?62?211111?21?1111111??????000????????????
?????????????????????0???2???????????????????????1?0?02?
'Ictidostoma_hemburyi'
1001100001010?01?0010111110111?0011101111011111?210111??????????????0??????110
0001011111?0?1010000000001000???????????????????0-00000-
'Trop_AZ_whaitsiid_SAM-PK-10990'
1001000001110?002001????????????00?1?111101111????01??????????????????????11000
11111111?0?1010?00001001000????????????????????-?001?-
'Hofmeyria_atavus'
1001{0,1}00001010101200{0,1}0111110111100111011110111110210111111?101210000?0001
11?111000{0,1}11111111001010100000{0,1}1000??????????????111??0-00010-
'Mirotenthes_digitipes'
1001000001010101200101111101111001110111?111111021??1111?101210000?000111?111
00001111111100101000?000011000110111111211??111110?-000{0,1}0-
'Ictidochampsa_platyceps'
1?0100010101?1012??111111?01111?0?110?1?????11?1?210111????01210?????0011?1?????
?????????????????????0?0?????0??????????????????????000?2-
'Viatkosuchus_sumini'
1001000101110100201111111011111001101162021111102101111010121 0??1?000111?11
10001?011111100101010?001??10?0????????????????????1000020
'Moschowhaitsia_vjuschkovi'
100100010111?10020111111??11??1001101262021111?2101?1??????????????0??????????
????????????????0??0??0?????0????????????????????1000020
'Theriognathus_microps'
1001{0,1}00101110100201111111001111100110{0,1}2620{1,2}11011{0,1}21111112110121000
1100011111{0,1}1000{0,1}10111111001010{0,1}00002011-01?10111112111?111110010--{0,1}-
0
'Ictidosuchus_primaevus'
?001101000001?01200101111011110001?1?121??111?1?211111110101211 01???0011???21
0000111?????0??11?101100?0?????1???????????????????-00000-
'Ictidosuchoides_longiceps'
1001101000001201200101111011110001110121011111121111110101211?10?001111?12
1000011111111000111{0,1}01100101000?????1?12?????1111?0-00000-
'Perplexisaurus'
???1?0?000???????00101?110011??100????11????11??021011??????????????????????000?0
00?1?11??0??1???0000010?110?????????????????????-00100?
'Ictidosuchops_rubidgei'
1001101000001?01200101111??11??000?1?01210{0,1}111110211111??????????????????????
????????????????????1101100??10?0?????????????????????0000{0,1}000
'Regisaurus_jacobi'
10011011000012012001101111011111?0011111310111111021111111110121 1?10100111?1?2
100001?111111??0011000100?01000?11111121111111111000{0,1}001
'Urumchia'
1??11??100?0????????????????????001???1310?11111?21?111????????????????????????1??0?
?????????????110001001 01000??????????????????????0?00?1?

'Choerosaurus_dejageri' ??01-011000010111?0111111-1111??10111?1?????1111??1????????????????????11???2110001011111101?1???011001010?0??0??11??11111?1?1????0101?

'Lycideops_longiceps' 1001101000001001100111111111111??10111014101111110211111????????1?????0??????210000101111110100?10001001{0,1}10?0??????????????????11100011

'Karenites_ornamentatus' ??011010000012011001{0,1}11111011110001111??1?01111102111101101012110101001111112110001011111101011???11001011?0???????????????????010001?

'Zambian_karenitid_NHCC_LB44' ???1101000001201100111111110111?0001111?41?211111?211111?1??01211????00111111211000101111110101????110010?020??????????????????010001?

'Tetracynodon_tenuis' ?0010010000010?210011?111-1?111?10111014??2111111211111111010?2?1010?00111???210000101111110?0?1200??00?0102?????????????????????1?0101?

'Tetracynodon_darti' 1001001000001012100111?111-1?111?1011101410211111121110111010121101010011111?121000010111111010112000??00?01020?2{0,1}11111211111111?11?01011

'Scaloposaurus_constrictus' 1001101000000 2?2?0010?11111?111?00111013101111111211111110101211?10100111???2100001011111101011?00?100101110?2???1112????111111?100100?

'Ericiolacerta_parva' ?001-011000002?210011?111-1?11??001100-51?2111?1121?111????0?2?100???0?11???2100001011111010111?0?2-0201110?210111??111????????0?01001

'Silphedosuchus' ?001-011000002??10011?111-1?1???00?1?0-51?211111121?1?1????????????????????21000?101?11??0?011??0?2-0201110???????????????????0?0100?

'Nothogomphodon' ?001100101010?01?0011??1??11???100?1?02310211111112111?1?????????????????????000?01111?11??0??110000100001110??????????????????01001001

'Ordosiodon' 1001100101010??????????????????0??1?125???11111121111????????????????????000000111111?10101100001001011110?2???1????????????101?01001

'Hazhenia' 100110000101011210011111101111110?110125??211111121111????012?100???0111?1?000000111111110101100?010010?110???????????????????100{0,1}011

'Antecosuchus' ????100101????????????????????????????25???????????11??????????????????????0101111?1?11??0?01????010010?1?0????????????????????1?00??1

'Bauria_cynops' 10011001010101122001111110111111001100251?2111111211111111101211000100111???0101111111111110001100001001011110??????12??????????01000001

'Traversodontoides' ???1100?01010?122001010110011 1?0001??0?5???11111121111 1????0?????????0???????10??1111?1???0??1?????10010?1?0???????????????????100000?

'Microgomphodon_oligocynus' 1001100101010 10{0,1}20010111101111100011002 51?21111121111111110121100010011 11?1010111111111111000110000100101110?21?01012????1?111101000001

```
        ;
ENDBLOCK;
```
